# Use of metamodels for rapid discovery of narrow bandgap oxide photocatalysts

**DOI:** 10.1016/j.isci.2021.103068

**Published:** 2021-08-30

**Authors:** Haoxin Mai, Tu C. Le, Takashi Hisatomi, Dehong Chen, Kazunari Domen, David A. Winkler, Rachel A. Caruso

**Affiliations:** 1 Applied Chemistry and Environmental Science, School of Science, STEM College, RMIT University, GPO Box 2476, Melbourne, VIC 3001, Australia; 2 School of Engineering, STEM College, RMIT University, GPO Box 2476, Melbourne, VIC 3001, Australia; 3 Research Initiative for Supra-Materials (RISM), Shinshu University, 4-17-1 Wakasato, Nagano 380-8553, Japan; 4 Office of University Professors, the University of Tokyo, 2-11-16 Yayoi, Bunkyo-ku, Tokyo 113-8656, Japan; 5 Monash Institute of Pharmaceutical Sciences, Monash University, Parkville, VIC 3052, Australia; 6 School of Biochemistry and Genetics, La Trobe University, Kingsbury Drive, 3042 Bundoora, Australia; 7 School of Pharmacy, University of Nottingham, NG7 2RD Nottingham, UK

**Keywords:** chemistry, catalysis, computational chemistry

## Abstract

New photocatalysts are traditionally identified through trial-and-error methods. Machine learning has shown considerable promise for improving the efficiency of photocatalyst discovery from a large potential pool. Here, we describe a multi-step, target-driven consensus method using a stacking meta-learning algorithm that robustly predicts bandgaps and H_2_ evolution activities of photocatalysts. Trained on small datasets, these models can rapidly screen a large space (>10 million materials) to identify promising, non-toxic compounds as candidate water splitting photocatalysts. Two effective compounds and two controls possessing optimal bandgap values (∼2 eV) but not photoactivity as predicted by the models were synthesized. Their experimentally measured bandgaps and H_2_ evolution activities were consistent with the predictions. Conspicuously, the two compounds with strong photoactivities under UV and visible light are promising visible-light-driven water splitting photocatalysts. This study demonstrates the power of machine learning and the potential of big data to accelerate discovery of next-generation photocatalysts.

## Highlights

• Stacking models predict bandgap and H_2_ evolution activity of oxide photocatalysts
• Models predict robustly across a wide range of material structures
• Models rapidly identify promising photocatalysts from 10 million materials
• Four compounds are synthesized and confirm predicted results

## Graphical abstract

**Figure:**
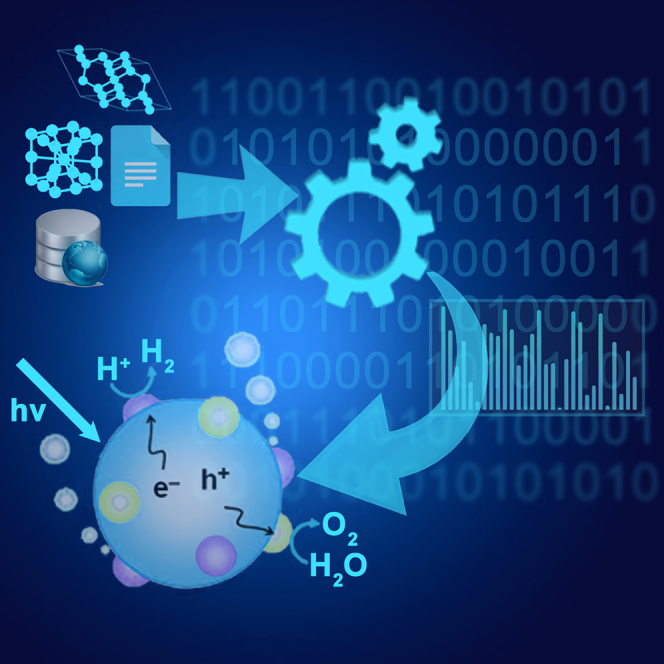

## Introduction

The ever-increasing need for energy and the clear environmental impact of fossil fuels in the 21^st^ century are driving a rapid move to renewable energy sources (Furlan and Mortarino, 2018; Meinshausen et al., 2009; Wang et al., 2015). Of these, solar energy is the most attractive because the sun provides free, renewable, abundant, and sustainable energy at a rate of ∼1 kW/m^2^ (Green and Bremner, 2017; Kannan and Vakeesan, 2016). An attractive way to store solar energy is through photocatalytic water splitting, producing hydrogen as clean chemical fuel from water by sunlight (Chen et al., 2017; Tachibana et al., 2012; Wang and Domen, 2020; Wang et al., 2015; Zhang et al., 2016). Since the discovery of the photocatalytic properties of TiO_2_ in 1972 (Fujishima and Honda, 1972), more than 140 types of inorganic photocatalysts have been discovered (Chen et al., 2019; Lee and Choi, 2018; Nursam et al., 2015; Pan et al., 2018; Wang and Domen, 2020; Wang et al., 2014, 2015, 2018, 2019b; Zhang et al., 2016). However, the large bandgaps or poor matching of the redox potentials of most photocatalysts results in low quantum efficiency and poor catalytic water splitting activity under visible light, hampering commercial applications (Kudo and Miseki, 2009). Clearly, much more effective photocatalysts are required to make solar hydrogen production commercially viable.

High-efficiency photocatalysts must satisfy two basic criteria. Firstly, although the free energy barrier for water splitting is 1.23 eV per electron, a bandgap of ∼2 eV is necessary for photocatalysts to trigger the water splitting reactions because of overpotentials, device operating voltage, and other losses (Zhu and Wang, 2017). Secondly, photocatalysts must have redox potentials that match the reaction, i.e., their conduction band minimum must be more negative than the H^+^/H_2_ potential (Wang and Domen, 2020). To discover materials that match these criteria, the traditional laboratory-based trial-and-error method has largely been used to discover suitable materials, albeit with low efficiencies and high cost. With the impressive developments in computing technologies and high-throughput combinatorial techniques, high-throughput screening based on density functional theory (DFT) has become feasible for investigating functional materials, including photocatalysts (Castelli et al., 2015; Davies et al., 2016; Wu et al., 2013; Zhang et al., 2018). A vast amount of data produced by these investigations has been collected in databases such as The Materials Project, providing electronic structures and thermodynamic information for materials researchers (Wang et al., 2016). Despite these achievements, the complexity of photocatalysts increases the computational cost of predicting the photocatalytic properties, and size of material space extends the screening time, resulting in low screening efficiency (Tabor et al., 2018). Until recently, even the most advanced high-throughput ab initio simulations were only tractable for a few thousand compounds, an infinitesimal fraction of the chemically accessible materials space (Davies et al., 2016). However, increases in computational hardware and algorithms have seen DFT calculations being performed for up to 1 million materials (Chanussot et al., 2021).

Advanced materials informatics and artificial intelligence provide an alternative means of tackling this dilemma (Butler et al., 2018; Jordan and Mitchell, 2015; Le et al., 2012; Sanchez-Lengeling and Aspuru-Guzik, 2018; Tabor et al., 2018). In particular, machine learning (ML) techniques have made rapid progress in the design of diverse materials such as organic-inorganic perovskites, metal-organic frameworks, phosphors, catalysts, and metallic glasses (Fanourgakis et al., 2020; Gómez-Bombarelli et al., 2016; Gu et al., 2019; Lu et al., 2018; Ren et al., 2018; Sun et al., 2020; Zhuo et al., 2018). ML models trained on a relatively small number of expensive first principles electronic structure calculations can predict a myriad of electronic, physical, and mechanical properties, such as formation energies, gas uptake, bulk and shear modulus, and bandgap (Chen et al., 2020; Himanen et al., 2019; Toyao et al., 2020). Furthermore, by applying feature engineering to these ML models, complicated feature-property relationships can be identified without any prior knowledge of the materials system (Tabor et al., 2018). For these reasons, ML models are becoming widely used for identifying new materials with bespoke properties from extremely large materials spaces.

Although ML techniques have been used to model existing photocatalyst properties, their use to discover new photocatalysts is still relatively uncommon (Can and Yildirim, 2019; Fathinia et al., 2016). There are two main problems in applying ML to photocatalyst design (Masood et al., 2019). One issue is that the structural diversity of photocatalytic materials is quite high. Most ML models are trained on small subsets of chemically similar materials, and these models have relatively small domains of applicability. They are therefore not capable of making accurate predictions of properties of materials in large photocatalyst databases. Another problem is that experimental photocatalysis data are relatively inconsistent. For example, H_2_ evolution rates are acquired in a range of reaction environments using different measurement protocols. The method of preparation can also affect photocatalytic properties. However, if all these experimental data are captured, they can be useful features for training ML models.

To address these challenges and provide fast computational screening of large databases to discover novel and efficient water splitting photocatalysts, here we describe a multi-step, target-driven modeling approach for discovery of narrow bandgap, non-toxic photocatalysts. Separate meta-learning ML models were generated for the bandgap and hydrogen evolution reaction (HER) activity, which are important for photocatalyst performance, using a dataset of chemically diverse photocatalysts. The meta-learning algorithm is a consensus method that uses a stacking algorithm to generate a metamodel from a set of base models. This approach generates robust and predictive bandgap and HER activity models that are superior to the base models. These models were used to search a material space of over one million unexplored materials. To provide initial validation of the utility of the method, four structurally diverse compounds predicted to have narrow bandgaps were synthesized. Subsequent experimental measurements of their bandgaps and HER activity showed excellent agreement with the model predictions. These results exemplify how accelerated design and discovery of novel water splitting photocatalysts may be achieved using ML techniques that require relatively modest computational and experimental resources.

## Results and discussion

**Figure 1:**
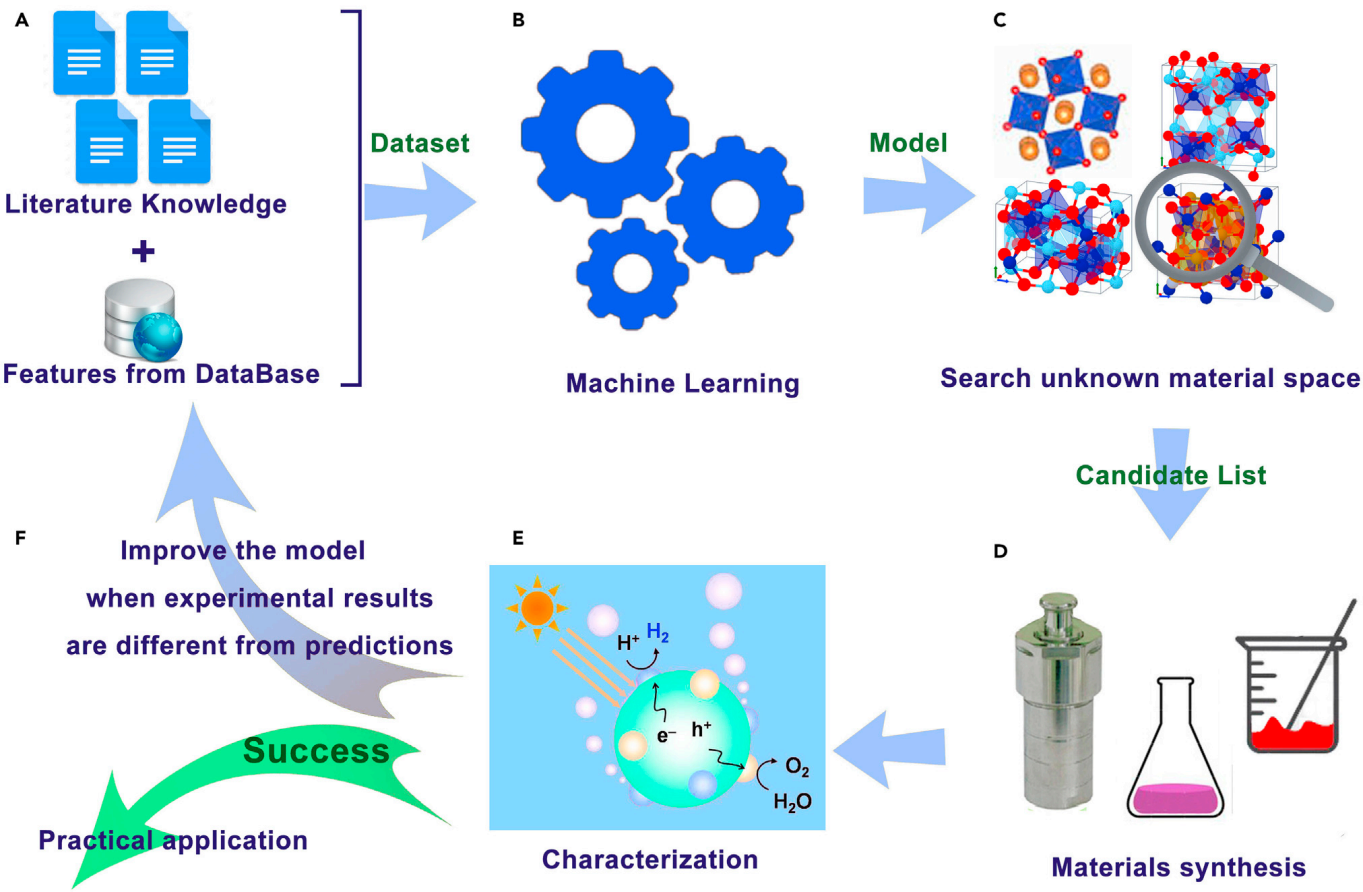
The workflow for target-driven narrow bandgap photocatalyst design (A) Chemical features and photocatalysis data from the literature were used to build the dataset. (B) Bandgap regression and HER activity classification ML models were trained on these. (C) The best ML models were used to scan unknown material space (∼10^6^ materials), identifying a list of candidates with optimal bandgaps and HER activity. (D and E) (D) These candidates were synthesized and (E) their bandgap and H_2_ evolution measured. (F) These new experimental data can be added to the dataset to improve subsequent models, closing the loop.

The dataset was randomly partitioned into a training set, 80% of the photocatalysts and a test set, 20% of the photocatalysts used to assess the prediction accuracy of the models. Models with the best performance on the test set were used to screen a large set of untested materials to identify a shortlist predicted to have optimal bandgaps and useful HER activity. These materials were subsequently synthesized and their electronic and photocatalytic properties measured. Materials identified by this computational screening paradigm constitute promising photocatalysts for commercial application. This multi-step, target-driven approach to design of narrow bandgap photocatalysts is depicted in Figure 1.

### Bandgap regression models

#### Machine learning base models

Most ML algorithms are capable of generating high-performance models that predict material properties and elucidate property-structure relationships (Butler et al., 2018; Chen et al., 2020; Gu et al., 2019; Tabor et al., 2018; Toyao et al., 2020). The largest performance differences are seen between linear and nonlinear models. Least absolute shrinkage and selection operator (LASSO), kernel ridge regression (KRR), artificial neural networks, support vector regression (SVR), random forest (RF), extra tree (EXT), and different types of gradient boosting regression (GBR) have been used to predict the bandgap of diverse compounds (Li et al., 2019, 2020a; Lu et al., 2018). Although these models could recapitulate the properties of photocatalysts well, two problems remain. Some models were trained on bandgaps calculated by DFT methods known to underestimate them (Wang and Pickett, 1983). Secondly, most models are trained on photocatalysts with limited chemical diversity. The small domains of applicability of these models compromises their abilities to predict photocatalytic properties for materials with a much wider range of structures such as perovskites, scheelites, spinels, and others (Wang and Domen, 2020).

We first generated ML models for photocatalyst bandgap prediction using 10 popular ML algorithms: RF, EXT, GBR, KRR, LASSO, ridge regression (Ridge), and SVR algorithms with radial basis function (SVR(rbf)), polynomial (SVR(poly)), and linear (SVR(linear)) kernels. Three metrics were used together to assess the performance of models: the coefficient of determination (R^2^), root mean squared error (RMSE), and mean absolute error (MAE). The latter two measures of dispersion are preferred over R^2^ values as they are not dependent on the number of data points and number of parameters in the model (Alexander et al., 2015). MAE values are less biased by one or two large outliers in the predictions than RMSE values.

The linear regression models (e.g. LASSO, Ridge, SVR(linear)) performed poorly in predicting the BG values for the test set (Table 1). The nonlinear RF, EXT, and GBR methods generated relatively accurate predictions of the test set bandgap values (MAE values of ∼0.3 eV for the test set). The performance of these models is illustrated graphically in Figure S3.

**Table 1:** Performance of various models on the bandgap predictions on the BG dataset

Models	R^2^	RMSE [eV)	MAE [eV]	t-value/p value
SVR(rbf)	0.94/0.81	0.26 ± 0.00/0.47 ± 0.00	0.19 ± 0.00/0.32 ± 0.00	3.72/0.021
SVR(poly)	0.61/0.58	0.61 ± 0.00/0.63 ± 0.00	0.51 ± 0.00/0.60 ± 0.00	4.91/0.008
SVR(linear)	0.46/0.38	0.73 ± 0.00/0.74 ± 0.00	0.62 ± 0.00/0.72 ± 0.00	5.77/0.004
LASSO	0.51/0.46	0.69 ± 0.00/0.71 ± 0.00	0.66 ± 0.00/0.72 ± 0.00	5.47/0.005
Ridge	0.51/0.45	0.69 ± 0.00/0.71 ± 0.00	0.67 ± 0.00/0.72 ± 0.00	5.57/0.005
KRR	0.92/0.82	0.46 ± 0.00/0.71 ± 0.00	0.23 ± 0.00/0.35 ± 0.00	5.05/0.007
RF	0.94/0.87	0.30 ± 0.02/0.37 ± 0.05	0.18 ± 0.01/0.28 ± 0.02	11.5/0.0003
EXT	0.94/0.88	0.28 ± 0.02/0.38 ± 0.05	0.20 ± 0.01/0.30 ± 0.02	9.55/0.0007
GBR	0.99/0.87	0.10 ± 0.02/0.35 ± 0.05	0.06 ± 0.01/0.30 ± 0.02	9.30/0.0007

Results are reported as training set/test set, RMSE and MAE are acquired from the average of 100 times training/testing. Paired sample t test is carried out between one base-model and STRBG model on RMSE, degrees of freedom is 5 and the pre-selected level of significance is 0.05.

#### Consensus metamodeling stacking algorithm approach

Despite the nonlinear models having good performance, none of them recapitulated the reported bandgaps to within the estimated experimental error of ±0.2 eV. The residual prediction error may result from the use of a small number of features and the wide diversity of materials in this dataset. In such cases, a hybrid (consensus) model may be more successful in predicting the photocatalytic properties of all materials in the dataset.

We employed a stacking algorithm that creates an ensemble of ML models to make more accurate predictions of the properties under study (Wolpert, 1992). Stacking aims to achieve generalization accuracy, rather than a learning accuracy, that is as high as possible. Unlike other consensus models, stacked generalization deduces the biases of the models with respect to a given training set. Stacking involves training a learning algorithm that combines the predictions of several other learning algorithms. The other (base) algorithms are trained first using the available data, then a combiner algorithm is trained on all predictions of the base algorithms as additional inputs to generate a metamodel that is more accurate than any of the base models. In theory, if an arbitrary combiner algorithm is used, then stacking can represent any type of ensemble learning (Chatzimparmpas et al., 2021; Ghasemian et al., 2020; Ma et al., 2018; Singh et al., 2019; Wang, 2018). Stacking is a possible solution to the problems posed by small datasets (Butler et al., 2018).

**Figure 2:**
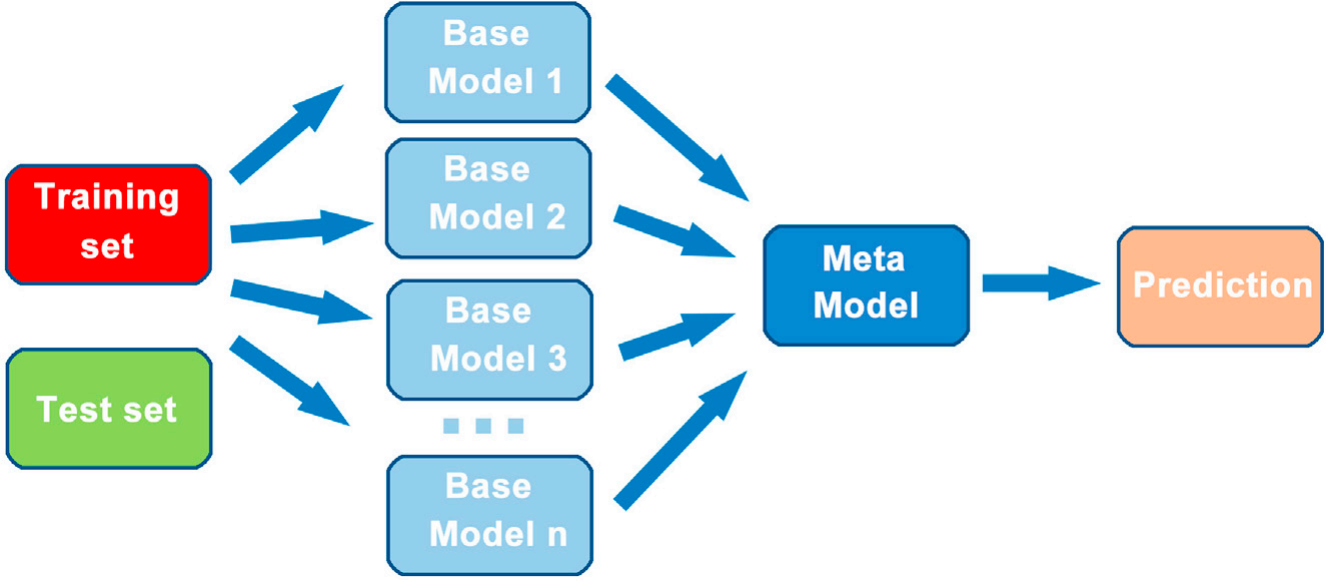
The architecture of the 2-level stacking model Each of the n base models consists of five weak models generated by 5-fold cross validation. The outputs of the base models for the training data were meta-features used to train a meta-model that finds the best combination of the base models for each input. The test set predictions of specific base models were averages (for regression) or votes (for classifiers) of the predictions from the weak models.

Figure 2 summarizes the architecture of the stacking model used in this work. It consists of two levels. At the base level, several ML algorithms are chosen, and each algorithm generates 5 models from the training datasets using 5-fold cross validation. The outputs of these base models are used as the features (meta-features) to train the metamodel in the next level. When the metamodel is trained by an appropriate algorithm, it results in the best combination of the base models for bandgap prediction of different types of materials (see Figure S4).

**Figure 3:**
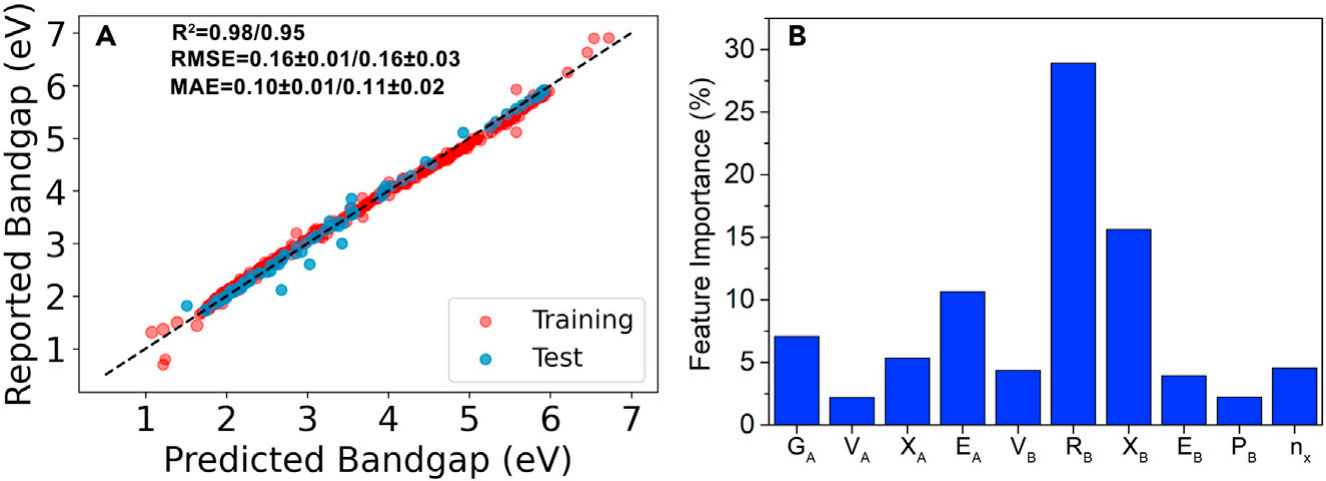
Performance of bandgap regression models (A) The bandgap of the photocatalysts in the BG dataset predicted by STR_BG_ model versus reported bandgap values. The R^2^, RMSE, and MAE were for the training set/test set. RMSE and MAE are acquired from the average of 100 times training/testing. (B) Relative importance of top 10 features from the GBR bandgap model.

Based on the preliminary ML models for the bandgap (Table 1), we selected six nonlinear algorithms, RF, EXT, GBR, KRR, SVR(rbf), and SVR(poly), as the base models. The poorly performing linear algorithms were not used. The final bandgap stacking model (STR_BG_) was trained using the SVR(rbf) algorithm. The R^2^, RMSE, and MAE of the STR_BG_ model was 0.97, 0.16 ± 0.03 eV, and 0.11 ± 0.02 eV, respectively, for the test set, close to the values for the training set prediction and the estimated experimental error. This suggested that the model was robust and not overfitted (Figure 3A). Comparison with the single ML models of bandgap predictions listed in Table 1 shows that the STR_BG_ model outperformed all other models (the best set RMSE value for the GBR models being 0.35 ± 0.05 eV). A paired t test showed that these RMSE values were different at the 99.7% confidence level.

The contributions of features to the bandgap model were estimated by GBR, the best performing base model algorithm for this dataset. As Figure 3B shows, the most important contributors to the bandgap model are R_B_, X_B_, and E_A_, reinforcing the importance of both components A and B on the bandgap. Although there was no simple relationship between features and bandgap, the model suggested that the oxides with optimal bandgap for water splitting are most likely to possess R_B_ in the range of 240–250 pm, X_B_ in the range of 1.5–2.2, and E_A_ > 520 kJ mol^−1^ (Figure S5). In other words, the oxides with optimal bandgap for water splitting are not likely to contain Al, Zr, Nb, or Bi at the B-site and an alkali metal element at the A-site of these oxides.

To evaluate the performance of the STR_BG_ model across a range of materials, 10 compounds not included in the BG dataset were extracted from the literature (Table 2) (Adak et al., 2020; Bai et al., 2012; Bouzidi et al., 2010; Maeda et al., 2020; Vavilapalli et al., 2018; Wheeler and Choi, 2018; Wu et al., 2012, 2017). These compounds are mainly used in electronic and magnetic devices and are not primarily photocatalysts. The predicted bandgap values from the STR_BG_ model and the experimental values from the literature are listed in Table 2. The MAE between the predicted value and the reported value was 0.17 eV, and the maximum error was −0.35 eV for Bi_6_Ti_3_Fe_2_O_18_. The table shows that the STR_BG_ metamodel is clearly the most reliable for predicting the reported bandgaps, with all predictions within 10%. This preliminary validation study suggests that the STR_BG_ model makes useful bandgap predictions for unknown compounds with diverse structures (see also Table S5).

**Table 2:** Predictions of the bandgap (eV) of the 10 unknown samples from base and metamodels

Material	STR_BG_	RF	GBR	EXT	KRR	SVR(rbf)	SVR (poly)	Reported bandgap
(Ba_0.5_Ni_0.5_)Bi_2_NbTaO_9_	2.72	3.16	3.14	2.92	2.98	2.69	2.94	2.55
Bi_2_Ti_4_O_11_	2.80	2.90	2.99	2.87	2.56	1.97	2.51	3.10
Bi_5_Ti_3_FeO_15_	2.25	2.43	2.15	2.17	2.07	1.67	1.98	2.03
Bi_6_Ti_3_Fe_2_O_18_	3.37	2.42	2.15	2.17	2.57	3.06	1.55	3.72
Ca_2_Fe_2_O_5_	2.22	2.43	2.00	2.14	2.22	2.06	2.39	2.10
LiVO_3_	3.34	3.15	3.41	2.92	3.38	2.88	3.56	3.30
KBiFe_2_O_5_	1.88	3.34	1.70	2.60	3.48	3.18	3.31	1.68
SrBi_2_Nb_2_O_9_	2.66	3.33	3.25	3.44	2.69	2.64	2.47	2.70
Sr_0.99_Bi_2.01_Nb_1.99_Ni_0.01_O_8.99_	2.50	3.34	3.24	3.35	2.98	2.71	2.32	2.45
Sr_0.91_Bi_2.09_Nb_1.91_Ni_0.09_O_8.91_	2.48	3.34	3.24	3.39	2.99	2.69	2.30	2.25
Predictions within 10%	10	1	4	3	3	3	1	

### H_2_ evolution classification models

H_2_ evolution experiments have been conducted using a range of conditions in different laboratories. For example, incident light, solutions, and co-catalysts used to boost H_2_ evolution vary, and the morphology, size, and surface area of the photocatalysts also differ. This makes it difficult to generate regression models of HER activity trained on the intrinsic properties of the photocatalysts alone: additional descriptors encoding differences in measurement protocols are required, but these are rarely recorded. To overcome this shortcoming, we used classification rather than regression methods to model the HER activity of photocatalysts in this work. We trained classification models on the same features used to train the bandgap regression models. We quantified the quality of predictions of the classification models using accuracy, F1 score, and the area under the receiver operating characteristic curve (AUC) metrics. The latter two metrics are suitable for unbalanced classification models where one class is more highly represented than the other. To improve the prediction accuracy, three ensemble algorithms, RF, EXT, and gradient boosting trees (GBTs), were applied, and three bagging models derived from 100 SVM classifiers with RBF, polynomial, and linear kernels were constructed. The results of these six classification models were used to train a stacking metamodel, using the EXT algorithm to form a stacking classifier (STC_H2_ I) (Figure 2). The results of modeling the HER activity using the 6 base algorithms and the stacking algorithm are summarized in Table 3.

**Table 3:** Performance of the six base and stacking metamodels on the H_2_ activity classification without bandgap descriptor (results are reported as training set/test set)

Models	AUC	Accuracy	F1 score
RF	0.98/0.83	0.94/0.81	0.96/0.81
GBT	0.98/0.80	0.93/0.82	0.96/0.83
EXT	0.96/0.82	0.93/0.81	0.96/0.81
Bagging (SVC-poly)	1.00/0.80	0.97/0.76	0.99/0.82
Bagging (SVC-rbf)	0.97/0.82	0.94/0.75	0.96/0.81
Bagging (SVC-linear)	0.97/0.82	0.94/0.77	0.96/0.83
STC_H2_ I	0.95/0.87	0.96/0.90	0.95/0.90

The 6 base algorithm models had almost identical AUC and F1 score accuracies of 82 ± 1%. Although the stacking model showed significantly better prediction accuracy (87-90%) for the test set compared with the six base models, its modest accuracy (Table 3 and Figure S6) suggested that additional chemical features may improve the accuracy of the H_2_ evolution activity metamodel. For H_2_ evolution to occur, the H^+^ in the solution must capture the photoelectrons at the surface of the photocatalyst. As this reaction is strongly affected by the band structure of the photocatalyst, we hypothesized that the bandgap may be a useful additional feature for the H_2_ evolution metamodel. Therefore, we added the experimental bandgaps or those predicted by the STR_BG_ model if experimental bandgaps were not available to the training set. The six base classifiers were retrained, and a second stacking classifier for H_2_ evolution activity (STC_H2_ II) was generated.

**Figure 4:**
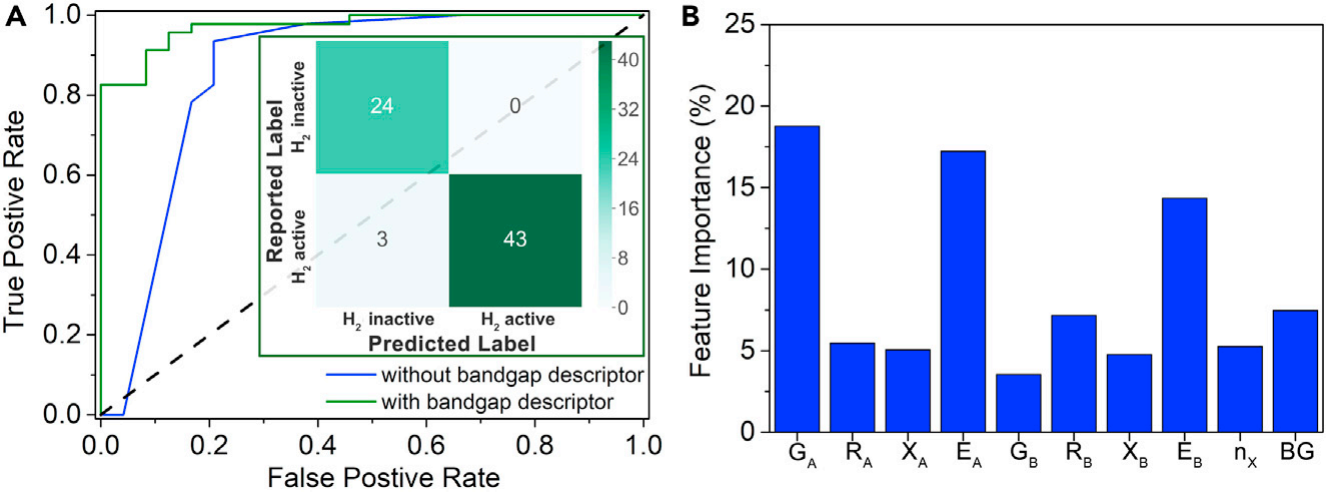
Performance of the H_2_ activity stacking classifier (A) ROC (receiver operating characteristic) curve of the stacking classifier with bandgap descriptor (green line) and without bandgap descriptor (blue line). Inset is the confusion matrix between true label (reported label) and predicted label using the stacking classifier with bandgap descriptor on the test set. (B) Relative importance of top 10 features from the average score evaluated by GBT and RF classifiers. BG denotes the bandgap descriptor.

Interestingly, as Table 4 and Figure S7 show, the test set prediction accuracy was only significantly improved for the three bagging models, with the other three algorithms providing slight improvement in accuracies compared to the models without bandgap descriptors. When considering both the F1 score and AUC metrics, RF, GBT, and the SVM classifier with an rbf kernel performed better than the other three models. However, the F1 score and AUC for predictions of the test set by the STC_H2_ II metamodel improved significantly to 96-97% (Figure 4A and Table 3). This again suggests the stacking algorithm is providing better model predictions than any of the base model algorithms.

**Table 4:** Performance of the six base- and stacking meta-models on the H_2_ activity classification with bandgap descriptor (results are reported as training set/test set)

Models	AUC	Accuracy	F1 score
RF	1.00/0.88	0.96/0.83	0.98/0.85
GBT	1.00/0.85	0.95/0.84	0.97/0.86
EXT	0.96/0.84	0.92/0.84	0.96/0.85
Bagging (SVC-poly)	1.00/0.85	0.99/0.82	0.92/0.83
Bagging (SVC-rbf)	1.00/0.87	0.97/0.87	0.98/0.86
Bagging (SVC-linear)	0.91/0.84	0.98/0.82	0.99/0.84
STC_H2_ II	0.99/0.97	0.98/0.96	0.98/0.96

The test set confusion matrix for the STC_H2_ II model, shown as an inset in Figure 4A, summarizes the accuracy of the active and inactive material predictions. The feature importance evaluated by the average score of RF and GBT (Figure S8) is plotted in Figure 4B. The relative importance of features to the GBT and RF models individually is shown in Figures S8A and S8B. The two algorithms provide similar rankings for the most important features. Bandgap is in the top five most important features (the top three being G_A_, E_A_, and E_B_), indicating its relevance to the models. Moreover, on further analysis, we found that a candidate photocatalyst is likely to have H_2_ activity if G_A_ < 6, E_A_ < 750 kJ mol^−1^, E_B_ < 700 kJ mol^−1^, and bandgap >3 eV (Figure S9). Considering also the results from the STR_BG_ model, photocatalytic oxides with optimal bandgaps may consist of Ca, Sr, Ba, Fe, Co, Ni, Cu, or Ag and rare earth elements at the A-site and Ti, V, Cr, Mn, Mo, or In at the B-site.

### HER model validation

Our studies have shown that the bandgap plays an important role in the modeling of HER active photocatalysts. It determines the range of incident light wavelengths that can be absorbed by the photocatalysts and has significant effects on the accurate prediction of H_2_ evolution activity via the STC_H2_ II model. The STR_BG_ metamodel is useful for identifying potential photocatalysts in hitherto unexplored materials that lack bandgap information. Taken together, the STC_H2_ II and STR_BG_ metamodels are useful for identifying small bandgap compounds with potential HER activity in visible light.

**Figure 5:**
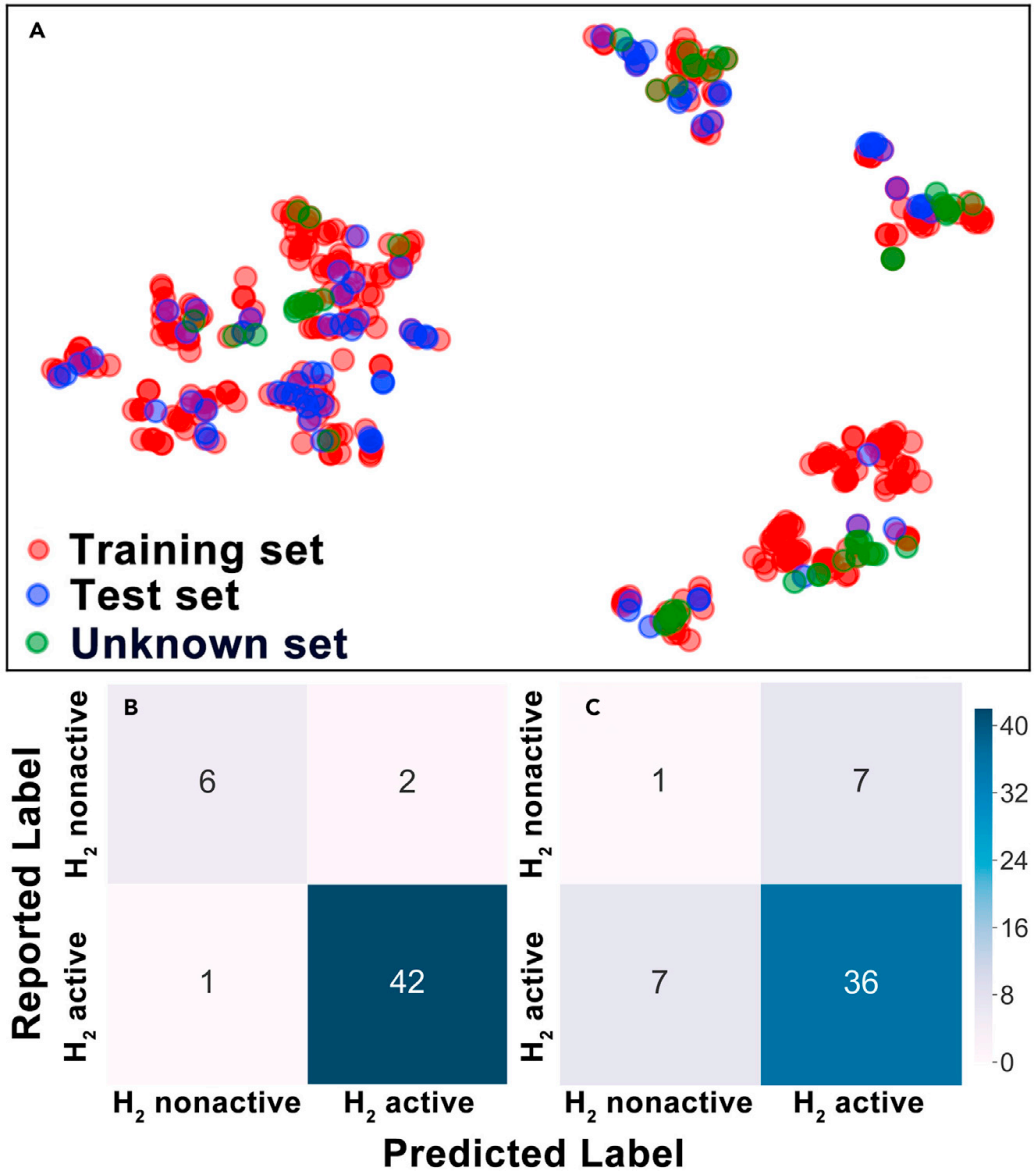
Performance of the combination of STR_BG_ model and SCT_H2_ II model on the unknown dataset (A–C)(A) Reduced two-dimensional feature space of the training set (red), test set (blue), and unknown set (green) obtained by the t-SNE algorithm. Confusion matrix between true label (reported label) and predicted label using the stacking classifier (B) with bandgap descriptor and (C) without bandgap descriptor on unknown dataset.

As a proof of concept, we collected 51 photocatalytic compounds from the literature where the bandgap was not reported or was uncertain (Table S6). These 51 compounds were not in the BG dataset and H_2_ dataset (Table S9 BG dataset related to STAR Methods and Table S10 H2 dataset related to STAR Methods), and thus, they are unknown to the models. Models are most useful in making predictions about new materials if they lie in or near its domain of applicability. Hence, we projected the training and test sets and these new materials from a 29-dimensional feature space to a 2D map using the t-SNE algorithm (Figure 5A) (Janet et al., 2019; van der Maaten and Hinton, 2008). This analysis suggests that materials in the new 51 dataset cover a similar feature space to that of the training set and test set. Thus, the STR_BG_ and STC_H2_ II metamodels should predict the bandgaps and HER activities with reasonable accuracy. We used the STR_BG_ model to predict the bandgap for the 51 new materials and employed this as an additional feature in the STC_H2_ II H_2_ evolution model. The confusion matrix in Figure 5B summarizes the H_2_ evolution prediction results for the new set of materials. Forty eight of 51 compounds were correctly classified (94% accuracy, similar accuracy to that for the test set, 96%). Only 37 compounds were correctly labeled when the bandgap was not included in the descriptor set (73% accuracy, Figure 5C). Again, the addition of bandgap as a descriptor increases the prediction accuracy of the STC_H2_ II model. This preliminary proof of concept shows that screening of narrow bandgap photocatalysts in a large material space using the metamodels is possible.

### Photocatalyst library virtual screening

**Figure 6:**
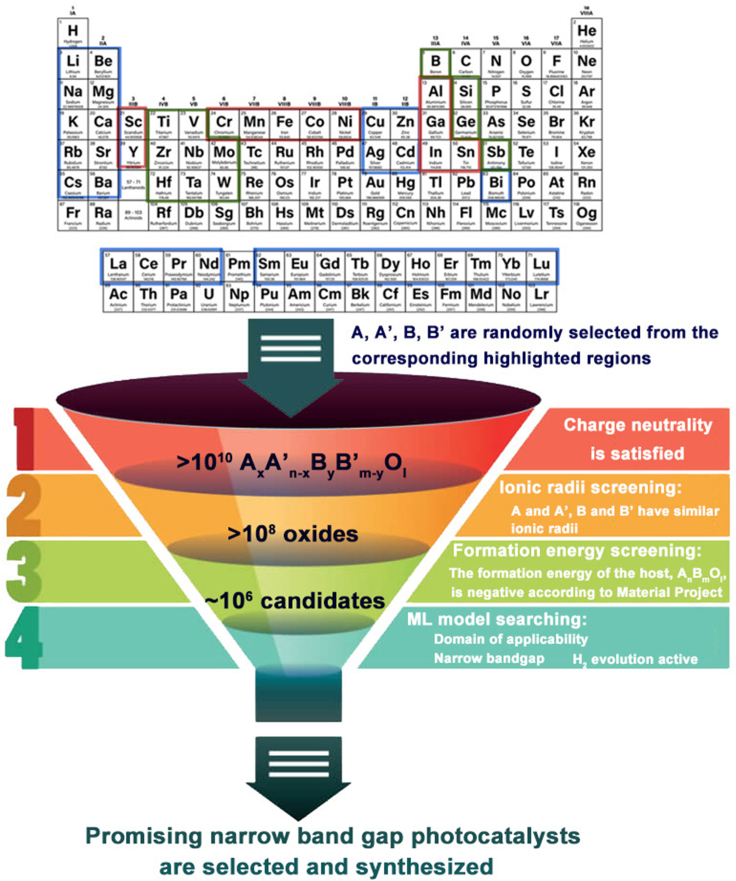
Schematic framework to search for novel photocatalysts (A_x_A'_n-x_B_y_B'_m-y_O_l_) based on ML models A, A′, B, and B′ were randomly selected from the corresponding highlighted regions in the periodic table, where the blue highlighted region is for A and A′, green highlighted region is for B and B′, while the elements highlighted red can be selected for A, A′, B, and B’. The combinations of these elements give rise to more than 10^10^ compounds. After screening for charge neutrality, stability, and ionic radii similarity, a candidate dataset of ∼10^6^ compounds was constructed. ML models were applied to predict the photocatalytic activities of this candidate dataset. Promising photocatalysts predicted to have narrow bandgaps and HER activity were identified for synthesis and characterization.

Given the promising results of the use of stacking metamodels discussed above, they were used to conduct a virtual screen of a large hypothetical library of potential photocatalytic materials to identify those with promising bandgaps and water splitting activities. The framework is illustrated in Figure 6. To ensure the library was close to, or within, the domain of applicability of the models, we divided the metal elements in the periodic table into three regions on the basis of the BG dataset (Table S9 BG dataset related to STAR Methods). Blue regions contain elements that are only found in the A site of materials in the training set, green regions denote elements found solely in the B site, and elements in the red regions are found in either the A or B site. Toxic and rare elements were excluded. An initial library of > 10^10^ electrically neutral compounds with the general formula A_x_A'_n-x_B_y_B'_m-y_O_l_ (x > 0, x ≥ n-x, y > 0, y ≥ m-y, l > 0) was established by combination of 40 A/A′ elements and 23 B/B′ elements. The stability of the host compound of A_x_A'_n-x_B_y_B'_m-y_O_l_, A_n_B_m_O_l_ (where n, m, l must satisfy the electrical neutrality of this compound) was estimated by their formation energy from the Materials Project Database (Jain et al., 2013). Only the materials with negative formation energy were considered to be stable, and the corresponding derivatives A_x_A'_n-x_B_y_B'_m-y_O_l_ were retained in the dataset. Finally, the number of compounds was reduced by ionic radii screening. This assumes that the A/A′ and B/B′ that can locate at the identical sites in a material have similar ionic radii. Thus, materials in which A/A′ and B/B′ have significantly different ionic radii were removed (e.g., Ba_0.5_Sr_0.5_TiO_3_ could pass the ionic radii screening but Ba_0.5_Be_0.5_TiO_3_ could not). These constraints reduced the number of screening candidates to ∼10^6^ and the photocatalytic activity of this set was predicted by the stacking metamodels. The STR_BG_ model was used to predict the bandgaps of the materials, and then, the STC_H2_ II model was used to predict their HER activity. Eventually, we selected the photocatalysts active in visible light (having bandgaps in the range 2.0–2.7 eV).

**Figure 7:**
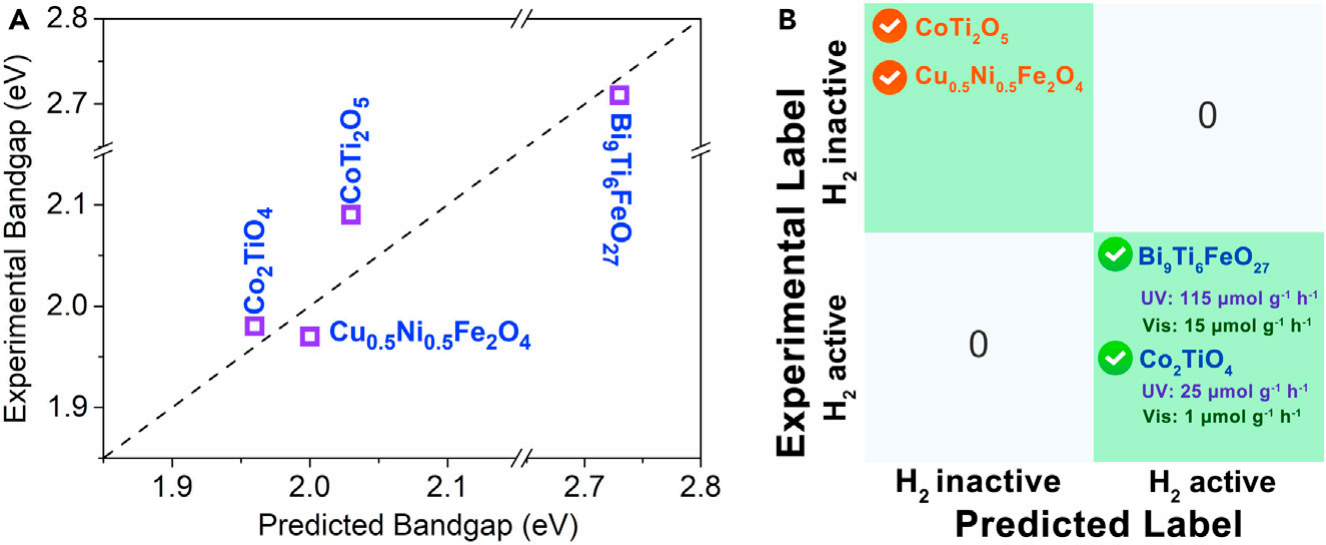
Photocatalysts obtained from ML model search (A) The bandgap of the 4 selected compounds predicted by the ML model versus experimental bandgap values. (B) Confusion matrix between true label (experimental label) and predicted label via the STC_H2_ II model.

A short list of ∼45,000 oxides was identified to be of visible light HER activity. Based on the novelty and reported synthesis methods (e.g., the selected candidates could be synthesized by the most commonly used methods, such as hydrothermal methods [<200°C], sol-gel methods [common additives, e.g., citric acid, are used and samples are calcined at a temperature <900°C], or solid-state reaction methods [<1500°C, in air and under normal pressure]), we selected 20 candidates from the short list, summarized in Table S7 and Figure S10. To verify the accuracy of this approach, two of the 20 candidates were synthesized, Bi_9_Ti_6_FeO_27_ and Co_2_TiO_4_. Two additional compounds in a similar feature space, CoTi_2_O_5_ and Cu_0.5_Ni_0.5_Fe_2_O_4_, that were predicted to not generate H_2_ but have bandgaps close to the optimal value (∼2 eV) were also synthesized as negative controls. The four compounds were pure phases (Figure S11). The experimentally determined bandgaps of these compounds were obtained from the measured UV-vis reflection spectra (Figure S12). These bandgaps were in excellent agreement with the predictions from the STR_BG_ model, with an MAE of 0.03 eV (Figure 7A and Table S8). Conspicuously, HER activity occurred for both Bi_9_Ti_6_FeO_27_ and Co_2_TiO_4_ under UV and visible illumination but was absent for CoTi_2_O_5_ and Cu_0.5_Ni_0.5_Fe_2_O_4_ under the same conditions (Figures 7B and S13). These results indicate that the predictions of the STR_BG_ model and STC_H2_ II metamodels were useful for virtual screening to identify new photocatalysts from large libraries of candidates.

Specific structural features of Bi_9_Ti_6_FeO_27_ and Co_2_TiO_4_ contributed to their HER activity compared to similar oxides. As a member of Aurivillius phase family (a form of perovskite represented by the general formulae (Bi_2_O_2_) (A_n−1_B_n_O_3n+1_)), Bi_9_Ti_6_FeO_27_ consists of alternate stacking of Bi_4_Ti_3_O_12_ and Bi_5_Ti_3_FeO_15_ layers. As a result, Bi_9_Ti_6_FeO_27_ showed H_2_ evolution activity similar to Bi_4_Ti_3_O_12_ which has a bandgap of 2.9–3.1 eV (Zhang et al., 2009)). However, it possessed a narrow bandgap (resulting from the Bi_5_Ti_3_FeO_15_ layer with bandgap of ∼2.0 eV (Wu et al., 2012) that does not exhibit H_2_ evolution activity). Co_2_TiO_4_ has a typical inverse spinel structure. Compared to the oxides of similar structure (e.g., CuM_2_O_4_ (M = Al, Cr, Mn, Fe, and Co), ZnMn_2_O_4_, ZnFe_2_O_4_), electron transfer efficiency was improved by the strong interaction between Co and Ti ions derived from the rigid metal-oxo-metal bridges (Li et al., 2020b), resulting in the HER activity in neutral environment. The metamodel approach identified these two compounds from millions of candidates without having structural information in advance. The H_2_ production from Bi_9_Ti_6_FeO_27_ and Co_2_TiO_4_ may be improved by optimization of the synthesis conditions, e.g., adjusting reaction time, temperature, and workup methods. Virtual screening of this type allows more time- and resource-intensive experimental and computational resources to be focused on the few most promising materials to further optimize and analyze them.

## Conclusions

A fast, multistep stacking metamodel method has been developed to discover narrow bandgap photocatalysts for water splitting. The metamodels for bandgap and HER activity were more accurate than any of the single base ML models used to train the metamodel. By rapidly screening a large, hypothetical materials library of >10^6^ candidate photocatalysts, 20 novel non-toxic examples were identified. Two materials, Bi_9_Ti_6_FeO_27_ and Co_2_TiO_4_, exhibited H_2_ evolution activity under both UV and visible illumination, in good agreement with the model predictions. Two other compounds with similar bandgaps CoTi_2_O_5_ and Cu_0.5_Ni_0.5_Fe_2_O_4_ did not generate H_2_ under illumination, also consistent with the metamodel predictions. The latent feature-property relationships captured by the ML models allowed the most important materials features to be identified. The most important features for the bandgap metamodel were radii, electronegativity, and ionization energy while for H_2_ evolution four features related to group, ionization energy, and bandgap play a key role. This ML-based, computational paradigm provides a rational basis for design of potential photocatalysts for water splitting.

As a relatively new strategy for material design, current ML modeling methods significantly accelerate discovery of materials with desirable properties. The construction of the ML models requires only relatively simple and accessible features from the literature or computational models rather than deep chemical and physical knowledge. These ML models require modest computing resources—screening of 10^6^ compounds can be completed within one hour, a great saving compared to other more rigorous, physics-based computational methods. These ML models allow experimental resources to be focused on the most promising materials, thus reducing the time for design, synthesis, and characterization. The improved generalization capabilities of the metamodels generated by the stacking algorithm allows investigation of a wider range of materials and improved discovery of novel photocatalytic materials, e.g., mixed anions such as oxynitrides (Pihosh  et al., 2020), oxysulfides (Wang et al., 2019a), and oxyhalides (Fujito et al., 2016) with optimal target properties. It is anticipated that an increasing number of functional materials with bespoke properties will be discovered and designed by ML modeling approaches in the future.

As we stated, there are inconsistencies in how H_2_ evolution activity is reported, restricting us to classification models. Given either metadata describing the differences in experimental measurements or a standardization of the experimental protocols, it should be possible to generate robust regression models that would be more useful for identifying the most active photocatalysts for water splitting.

### Limitations of the study

We built the bandgap regression model and H_2_ evolution activity classification model based on small datasets using the stacking algorithm. Although the performance of these models was good, the construction and optimization of stacking models can be complicated due to the choice of base models and the optimization of the hyperparameters of each base model. Individual models, such as RF, EXT, and GBR, showed relatively good predictions on the test set, but their generalization ability was poor, probably because of insufficient training data and choice of features. Ideally, given sufficient training data and optimal features, most ML modeling algorithms may have similar predictive abilities to the stacking models.

An additional limitation is the choice of the bandgap for the most commonly reported structure rather than considering all structures and bandgaps. Accommodating these would require a method for encoding the different structural types.

## STAR★Methods

### Key resources table

**Table:** 

REAGENT or RESOURCE	SOURCE	IDENTIFIER
**Chemicals, peptides, and recombinant proteins**		

Bismuth(III) nitrate pentahydrate	Aldrich	CAS: 10035-06-0
Iron(III) nitrate nonahydrate	Aldrich	CAS: 7782-61-8
Titanium(IV) isopropoxide	Aldrich	CAS: 546-68-9
Ethanol	Supelco	CAS: 64-17-5
Sodium Hydroxide	Chem-supply	CAS: 1310-73-2
Copper(II) nitrate trihydrate	Aldrich	CAS: 10031-43-3
Nickel(II) nitrate hexahydrate	Aldrich	CAS: 13478-00-7
Cobalt(II) nitrate hexahydrate	Aldrich	CAS: 10026-22-9

**Software and algorithms**		

scikit-learn	Open-source	https://scikit-learn.org/stable/
Mlxtend	Open-source	http://rasbt.github.io/mlxtend/

**Other**		

Bruker D4 Endeavor powder X-ray diffractometer	Bruker	https://www.brukersupport.com/ProductDetail/732
Agilent Cary 5000 spectrophotometer	Agilent	https://www.agilent.com/en/product/molecular-spectroscopy/uv-vis-uv-vis-nir-spectroscopy/uv-vis-uv-vis-nir-systems/cary-5000-uv-vis-nir
Closed-circulation system	Makuhari	AU-306-T02-S2

### Resource availability

#### Lead contact

Further information and requests for resources should be directed to Rachel Caruso (rachel.caruso@rmit.edu.au).

#### Materials availability

There are restrictions to the availability of the photocatalysts as we do not stock excess synthesized materials. The materials can be prepared as described in the Experimental Procedure.

### Method details

#### Model training data

The performance of ML models depends on the size and diversity of the training data (Butler et al., 2018; Chen et al., 2020; Le et al., 2012). Two datasets of diverse photocatalytic metal oxides were compiled: one used to train regression models to predict bandgaps, the other for H_2_ evolution classification model training. 489 oxides with experimental optical bandgap values (Table S9 BG dataset related to STAR Methods) were extracted from the literature, of which 380 oxides have binary H_2_ evolution data (Table S10 H2 dataset related to STAR Methods, active = H_2_ evolution, inactive = no H_2_ evolution). The two datasets have been included as supplemental information. The BG dataset (Table S9 BG dataset related to STAR Methods) was composed of oxides with diverse structures that had been reported to be photocatalytically active, including perovskite (46%), pyrochlore (15%), ilmenite (8%), scheelite (10%), spinel (5%), trirutile (2%), brannerite (2%), delafossite (2%) and others, as can be seen in the dataset (supplemental information). Photocatalysts likely to split water using visible light activity required bandgaps of 2.0 – 2.7 eV. 33% of the compounds in the BG dataset (Table S9 BG Dataset related to STAR Methods) were in this range.

As the preparation method can influence HER activity, we defined materials as HER active if at least one article reported photocatalytic H_2_ evolution, and inactive when its reported conduction band minimum is positive and no article reported photocatalytic H_2_ production. All oxides can be represented by a general formula A_x_A’_n-x_B_y_B’_m-y_O_l_ (x>0, x≥n-x, y>0, y≥m-y, l>0), where A/A’ and B/B’ are elements with similar properties that always reside in identical sites of a compound. Here, we classified the metal elements of the oxides as A or B according to their structure. For example, in perovskites (ABO_3_) a B element is a smaller six-coordinate ion, and an A element is a larger twelve-coordinate ion. In spinels (AB_2_O_4_) cations A and B occupy the octahedral and tetrahedral sites in the lattice, respectively. The sequences of A/A’ and B/B’ were determined following these rules: 1) the amounts of A (n_A_) and B (n_B_) were larger than A’ (n_A’_) and B’ (n_B’_); 2) when n_A_ = n_A’_ or n_B_ = n_B’_, the periods of A and B were smaller than A’ and B’; 3) when both the amount and periods of A/A’ and B/B’ were equal, the groups of A (G_A_) and B (G_B_) were smaller than A’ (G_A’_) and B’ (G_B’_). Given that the bandgaps of some compounds vary due to differences in structure, only the bandgap for the most frequently reported structure was used in the photocatalysis data set. For example, only the monoclinic BiVO_4_ phase was considered (bandgap 2.4 eV), rather than the tetragonal and orthorhombic BiVO_4_ forms.

The cation elements of these oxides cover a large part of the periodic table (40 types of A and A’ elements, and 30 types of B and B’ elements). Thus, ML models trained on these datasets should generalize well to new materials.

#### Features and feature selection

The materials in the dataset were described by chemical features (descriptors) obtained from materials handbooks and databases. The choice of features is important as it is one of the main factors (along with data quantity, quality, and diversity) determining model quality. Although many features may correlate with the target properties, the number of features must be limited to avoid overfitting and degradation of model predictivity by the presence of low relevance features (noise). Large numbers of descriptors also increase the complexity of the model, increasing the computation expense and compromising model predictivity and interpretability (Le et al., 2012). Feature selection was therefore employed to remove redundant and uninformative features, and this relevant subset of features was used to train and test a series of ML models.

Simple, basic atomic and physicochemical features were calculated for the dataset (Lu et al., 2019). Each component of the photocatalysts (A, A’, B, B’) was described by 14 features obtained from the periodic table, materials handbooks, and material databases (Table S1) (Li et al., 2019, 2020a; Lu et al., 2019; Rajan et al., 2018). To refine the number of features to the most relevant subset the full descriptor set of 57 features (56 features related to A, A’, B and B’, while n_x_ is the amount of O) was subjected to feature selection. To ensure the derived ML models can be extended to mixed-anion photocatalysts (e.g., A_x_A’_n-x_B_y_B’_m-y_O_l_X_l-z_), the anion elements O and X can also be described by the same 14 features. In this work, however, we focussed on the photocatalytic oxides.

The initial set of 57 features were pruned by removing those with low variance, and those highly correlated with other features using Pearson correlation coefficients (Figure S1). The remaining features were ranked by the GBR algorithm according to their relative importance and the least important feature removed. This process is repeated and the model score (R^2^) of trained ML model at each step used to identify the feature subset with the best performance (Figure S2 and Table S2). After this process, the optimal subset contained 29 features. This optimal feature set consists of the group number in the periodic table (G), Van der Waals radii (R), valence (V), electronegativity (X), ionization energy (E), polarizability (P) for all metal elements, and the mole fraction (n) of metal elements and oxygen.

#### Machine learning

The datasets are randomly split into a training set (80%) and test set (20%) (Le et al., 2012). The supervised ML regression models for predicting the bandgap of photocatalysts were trained on the BG training set (391 photocatalysts, 80% of the 489 photocatalysts in BG dataset). The supervised classification models for predicting HER activity of photocatalysts were trained on the H_2_ training set (304 photocatalysts, 80% of the 380 photocatalysts with HER activity reported from the literature). The test sets were only used for testing the performance of the ML models, and were not involved in any training processes. The base ML models were generated by these algorithms: SVR; SVC; RF; LASSO; ridge regression, EXT; GBR; GBT; and Bagging from the open-source scikit-learn package. The base models were optimized by grid searching methods (5-fold cross validation on training set, the optimized hypermeters were listed in Tables S3 and S4), and then 5-fold cross validation was conducted on the training set to generate five models for each algorithm for the base level of the stacking model (Figure 2). The stacking algorithm meta-models were constructed using the open source mlxtend package. Again, for the stacking models, the combination of the base models, and selection of the meta-model, were also optimized by grid searches (5-fold cross validation on training set, the optimized hypermeters were listed in Tables S3 and S4).

#### Photocatalyst synthesis

The four compounds of interest identified by the ML models were Bi_9_Ti_6_FeO_27_, Co_2_TiO_4_, CoTi_2_O_5_, and Cu_0.5_Ni_0.5_Fe_2_O_4_. Bi_9_Ti_6_FeO_27_ and Cu_0.5_Ni_0.5_Fe_2_O_4_ were synthesized by hydrothermal methods, while Co_2_TiO_4_ and CoTi_2_O_5_ were synthesized by co-precipitation.

*Bi*_*9*_*Ti*_*6*_*FeO*_*27*_. Bi(NO_3_)_3_∙5H_2_O (2.0 mmol, Aldrich, 98%) and Fe(NO_3_)_3_∙9H_2_O (0.22 mmol, Aldrich, 98%) were dissolved in MilliQ water (30 mL), while titanium(IV) isopropoxide (1.33 mmol, Aldrich, 97%) was quickly added to ethanol (10 mL, Supelco, 99%). These two solutions were then mixed, and the pH value of the solution was adjusted to 11 with the dropwise addition of NaOH (2.5 M, Chem-supply, 98%). After 30 min vigorous stirring, the as-prepared mixture was transferred into a 50 mL Teflon-lined autoclave and heated at 180°C for 24 h. After the solvothermal treatment, precipitates were separated by centrifugation, and washed with deionized water and ethanol 3 times, followed by drying at 60°C in air overnight. The dried samples were then calcined in a furnace at 700°C for 15 min in air.

*Cu*_*0.5*_*Ni*_*0.5*_*Fe*_*2*_*O*_*4*_. Cu(NO_3_)_2_∙3H_2_O (1.0 mmol, Aldrich, 98%), Ni(NO_3_)_2_∙6H_2_O (1.0 mmol, Aldrich, 98.5%), and Fe(NO_3_)_3_∙9H_2_O (2.0 mmol, Aldrich, 98%) were dissolved in MilliQ water (30 mL). The pH value of the solution was adjusted to 11 with the dropwise addition of NaOH (2.5 M, Chem-supply, 98%). After 30 min vigorous stirring, the as-prepared mixture was transferred into a 50 mL Teflon-coated autoclave and heated to 180°C for 24 h. After the hydrothermal treatment, precipitates were separated by centrifugation, and washed with deionized water and ethanol 3 times, followed by drying at 60°C in air overnight.

*Co*_*2*_*TiO*_*4*_. Co(NO_3_)_2_∙6H_2_O (2.0 mmol, Aldrich, 98%) was dissolved in MilliQ water (30 mL), while titanium(IV) isopropoxide (1.0 mmol, Aldrich, 97%) was quickly added into ethanol (10 mL, Supelco, 99%). These two solutions were then mixed, and the pH value of the solution was adjusted to 11 with the dropwise addition of NaOH (2.5 M, Chem-supply, 98%). After 60 min vigorous stirring, precipitates were separated by centrifugation, and washed with deionized water and ethanol 3 times, followed by drying at 60°C in air overnight. The dried samples were then calcined in a furnace at 850°C for 4 h in air.

*CoTi*_*2*_*O*_*5*_. Co(NO_3_)_2_∙6H_2_O (1.0 mmol, Aldrich, 98%) was dissolved in MilliQ water (30 mL), while titanium(IV) isopropoxide (2.0 mmol, Aldrich, 97%) was quickly added into ethanol (10 mL, Supelco, 99%). These two solutions were then mixed, and the pH value of the solution was adjusted to 11 with the dropwise addition of NaOH (2.5 M, Chem-supply, 98%). After 60 min vigorous stirring, precipitates were separated by centrifugation, and washed with deionized water and ethanol several times, followed by drying at 60°C in air overnight. The dried samples were then calcined in a furnace at 1150°C for 6 h in air.

#### Characterization

The crystalline phases of the synthesized photocatalysts were investigated using a Bruker D4 Endeavor powder X-ray diffractometer (XRD) with Cu Kα radiation. The samples were scanned from 10 to 75° in 2θ at a step size of 0.02°. UV−visible-near infrared (UV−vis-NIR) reflection spectra were measured on an Agilent Cary 5000 spectrophotometer equipped with a Harrick Praying Mantis accessory. The photocatalytic H_2_ evolution was carried out with 0.2 g photocatalyst (loading 0.1 wt% Pt as cocatalyst by photodeposition) suspended in a 200 mL aqueous methanol solution (10 vol%) in a Pyrex glass reaction cell. The reaction cell was connected to a gas-closed system with a gas-circulated pump. A 300-W Xe arc lamp was employed for the light source of the photocatalytic reaction. The reaction system was degassed by evacuation and then filled with 10 kPa Ar. During the visible-light reaction (400 nm < λ < 800 nm), a L42 cut-off filter was used to remove UV light.

## Data Availability

The data for this study are available within the article and the supplemental information, or from publicly accessible databases (The Materials Project). Any additional information required to reanalyze the data reported in this paper is available from the lead contact upon request.

## Supporting Citations

The following references appear in the supplemental information: Anderson et al., 2019; Asai et al., 2014; Castelli et al., 2013; Goto et al., 2018; Ishihara et al., 1999; Ishii et al., 2004; Khanahmadzadeh et al., 2015; Kim et al., 2005; Konta et al., 2003; Maeda, 2014; Masanobu et al., 2008; Ogura et al., 1997; Sahoo and Maggard, 2013; Sakata et al., 2016; Sato et al., 2003; Subbanna et al., 1990; Velinov et al., 2013; Yang et al., 2009; Yang et al., 2017; Yuan et al., 2010; Zhang et al., 2010; Zhang et al., 2012; Zhou et al., 2017.

## Supplemental information

Document S1. Figures S1–S13 and Tables S1–S8

Table S9. BG dataset, related to STAR Method s

Table S10. H2 dataset, related to STAR Method s

## Footnotes

Supplemental information can be found online at https://doi.org/10.1016/j.isci.2021.103068.
