# Supplementary material for: Use of metamodels for rapid discovery of narrow bandgap oxide photocatalysts
Source: iScience. 2021 Aug 30;24(9):103068. doi: 10.1016/j.isci.2021.103068 (PMC8455646; doi:10.1016/j.isci.2021.103068)
Supplement: Document S1. Figures S1–S13 and Tables S1–S8 [file mmc1.pdf]

iScience, Volume *24*

Supplemental information

Use of metamodels for rapid discovery

# of narrow bandgap oxide photocatalysts

Haoxin Mai, Tu C. Le, Takashi Hisatomi, Dehong Chen, Kazunari Domen, David A. Winkler, and Rachel A. Caruso

Table S1. Fourteen initial features for each element of the photocatalysts with description, related to Star Methods.

Features	Description
G	Group number of periodic table
V	Valence
R	Van der Waals radius
X	Electronegativity
E	Ionization energy
P	Polarizability
M	Atomic mass
MV	Molar volume
AR	Empirical atomic radius
EA	Electron affinity
BP	Boiling point
MP	Melting point
EF	Enthalpy of fusion
n	Amount

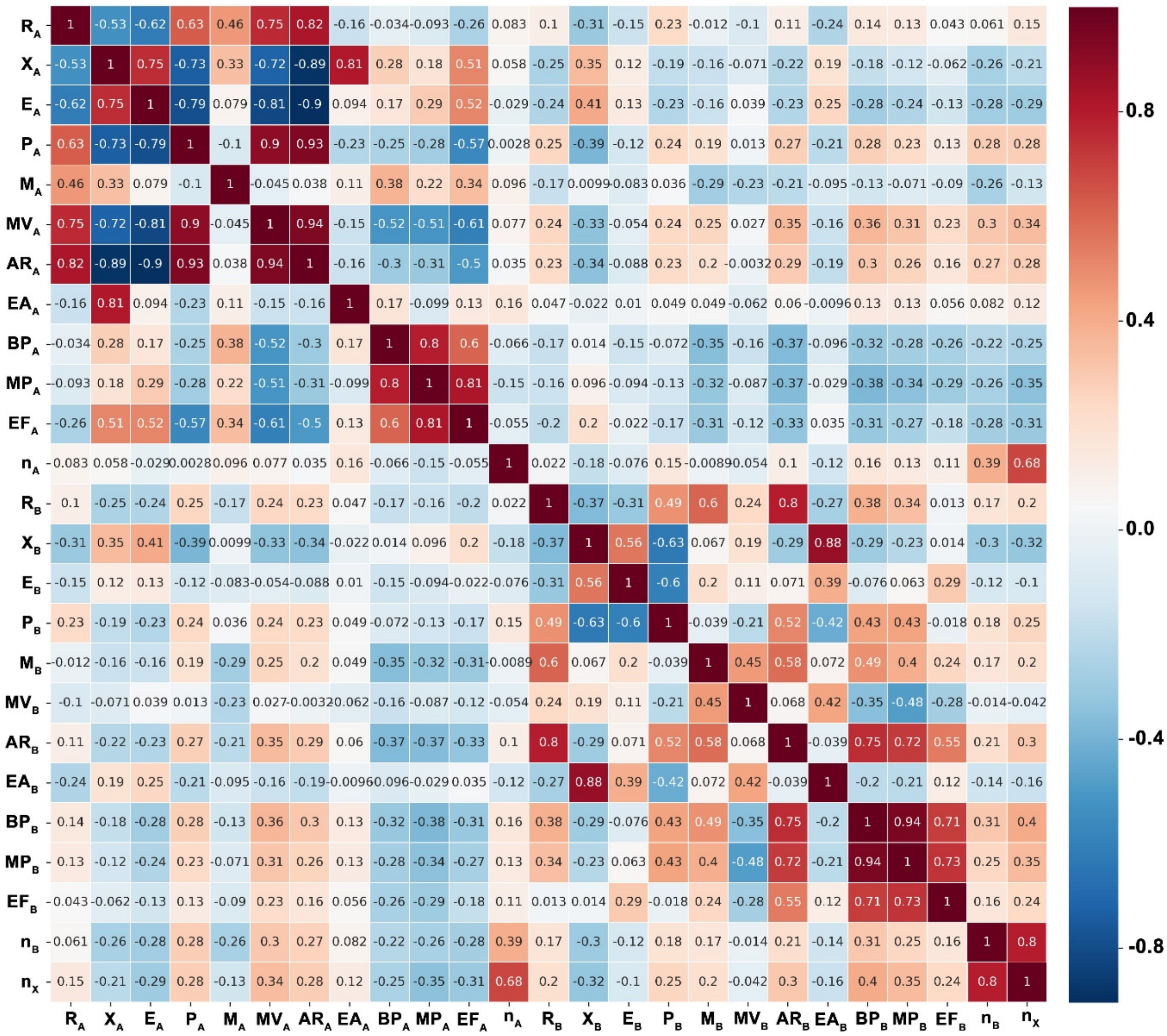

Figure S1. The Pearson correlation coefficient heat map of the features having continuous values and roughly following normal distribution, related to Star Methods. 25 features are shown in this figure, including 12 features for element A: Van der Waals radii (R_A), electronegativity (X_A), ionization energy (E_A), polarizability (P_A), atomic mass (M_A), molar volume (MV_A), atomic radii (AR_A), electron affinity (EA_A), boiling point (BP_A), melting point (MP_A), enthalpy of fusion (EF_A) and amount (n_A); 12 features for the element B: Van der Waals radii (R_B), electronegativity (X_B), ionization energy (E_B), polarizability (P_B), atomic mass (M_B), molar volume (MV_B), atomic radii (AR_B), electron affinity (EA_B), boiling point (BP_B), melting point (MP_B), enthalpy of fusion (EF_B) and amount (n_B), and the amount of element X (n_X).

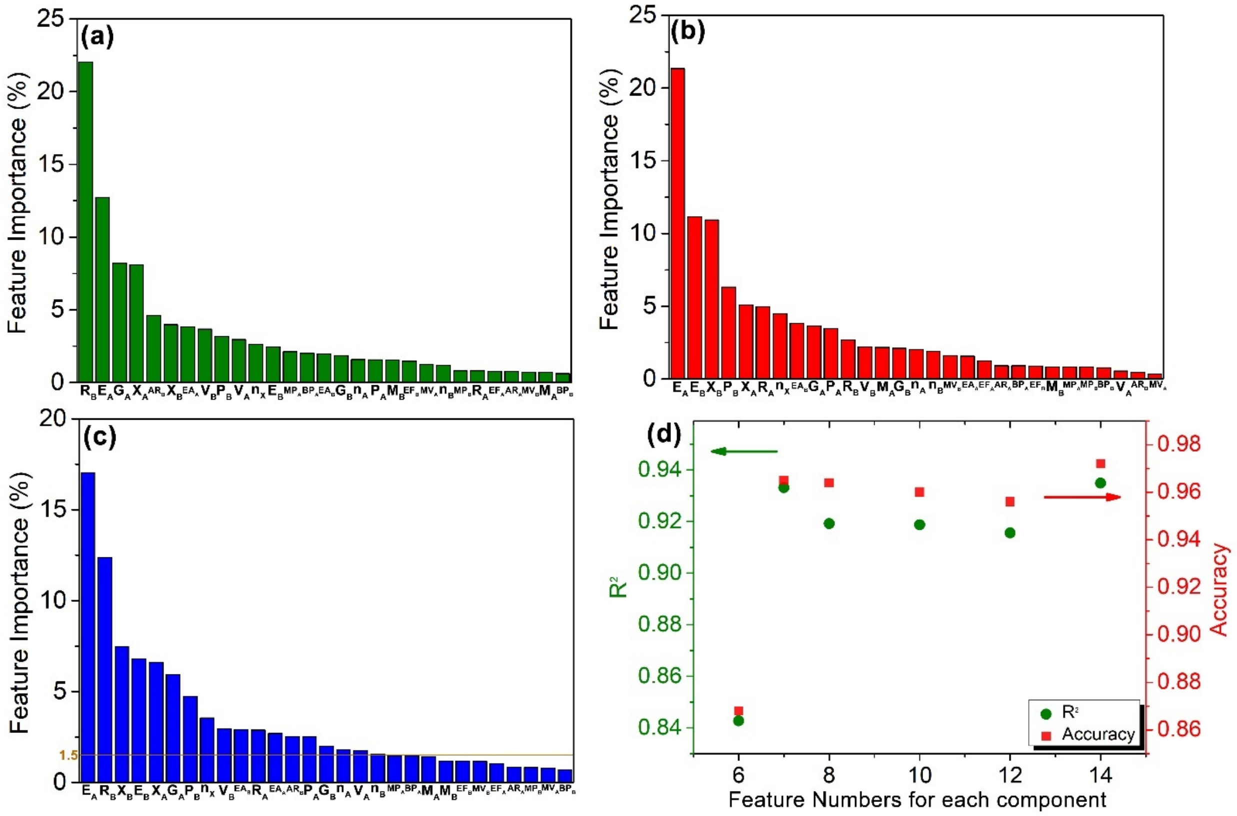

Figure S2. Relative importance of 29 features evaluated by the gradient boosting model trained on (a) BG dataset and (b) H2 dataset. G_A and V_A denote the group number in the periodic table and the valence of element A, respectively, and G_B and V_B denote the group number in the periodic table and the valence of element B, respectively. (c) The average of the relative importance in Figure S2a and Figure S2b. 1.5 is highlighted in the image as an importance percentage of 1.5 or below had little effect on model performance. (d) R^2 of the gradient boosting regression model (green dots) and accuracy of gradient boosting classification model (red square) versus feature numbers of each component. These figures are related to Star Methods

Table S2. Feature coefficients estimated by LASSO, related to Star Methods.

Features	Coefficients	Features	Coefficients	Features	Coefficients
G_A	-0.31	G_B	0.11	n_X	0.14
V_A	0.11	V_B	0.023		
R_A	-0.29	R_B	0.30		
X_A	-0.33	X_B	0.30		
E_A	0.073	E_B	-0.21		
P_A	0.14	P_B	-0.26		
M_A	0	M_B	0		
MV_A	0	MV_B	0		
AR_A	0.053	AR_B	0.13		
EA_A	-0.0044	EA_B	0.015		
BP_A	0	BP_B	0		
MP_A	0	MP_B	0		
EF_A	0	EF_B	0		
n_A	-0.21	n_B	0.23		

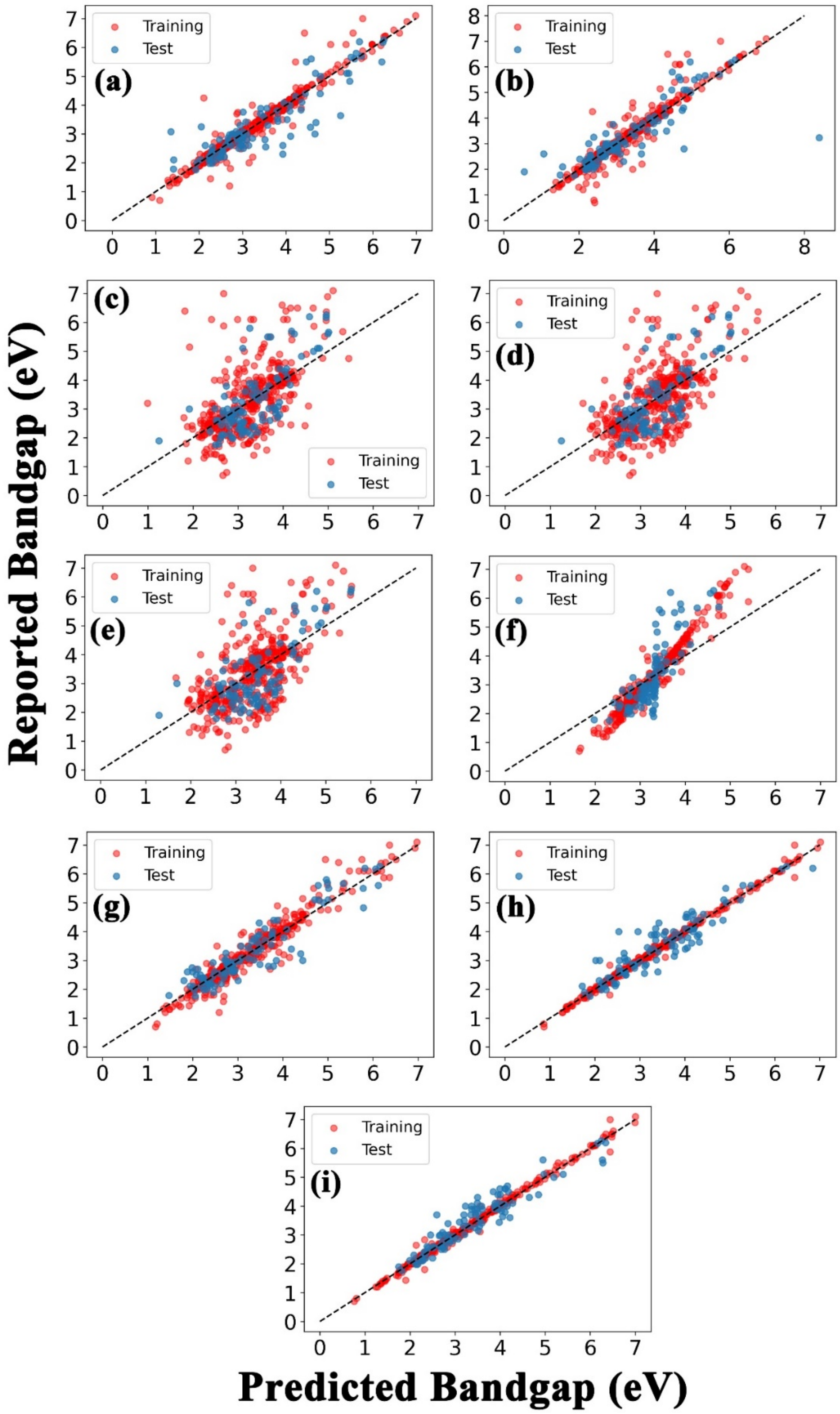

Figure S3. The bandgap of the photocatalysts in the BG dataset predicted via (a) SVR(rbf), (b) SVR(poly), (c) SVR(linear), (d) LASSO, (e) Ridge Regression, (f) KRR (kernel=rbf), (g) RF, (h) EXT, and (i) GBR versus reported bandgap values, related to Table 1.

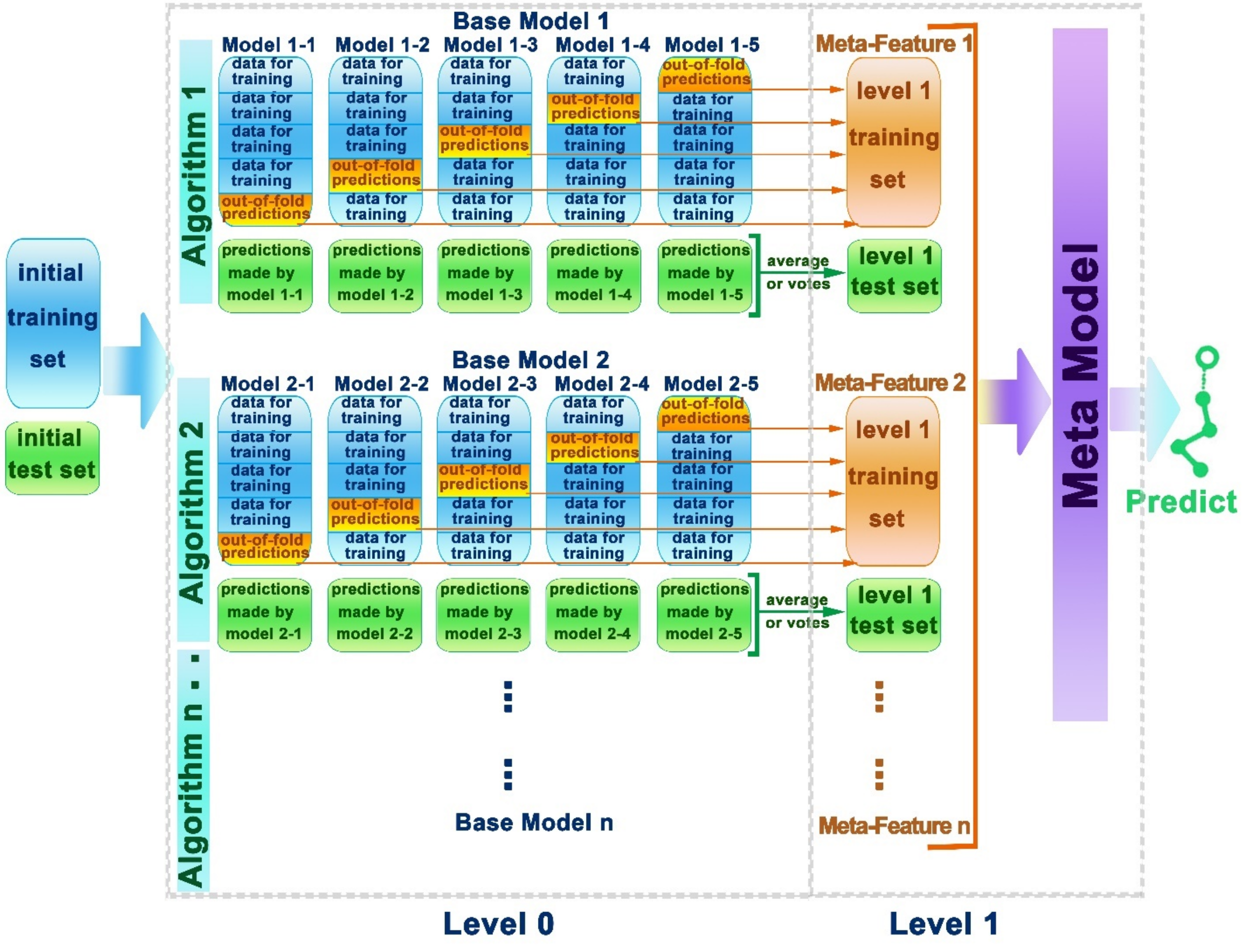

Figure S4. The detailed architecture of a stacking model with two levels, related to Figure 2. All the models would only be trained on data from the training set, while the test set, which is completely separated from the training set, would only be used to test the performance of the models and would never be involved in any training processes. 5-fold cross validation was carried out on the training set to build the base models. That is, the initial training set was divided into five subsets (e.g., T1-1 to T1-5). Weak model 1-1 was trained by the data in T1-1 – T1-4 (via Algorithm 1), and T1-5 was the out-of-sample data for model 1-1. After training, model 1-1 was used to predict T1-5 (T1-5 is a validation set for weak model 1-1), which was called out-of-fold predictions. Note that T1-1 – T1-5 were all from training set and test set data was not involved in the 5-fold cross validation. Similarly, weak model 1-2 was trained by the data in T1-1, T1-2, T1-3 and T1-5 then T1-4 would generate the out-of-fold predictions. Consequently, base model 1 consisted of five weak models (models 1-1 to 1-5) and would have five out-of-fold predictions. The five out-of-fold predictions were compiled as the meta-feature 1 in the level-1 training set. That is, meta-feature 1 in level-1 training set consisted of out-of-fold predictions OP1-1 (made by model 1-5), OP1-2 (made by model 1-4), OP1-3 (made by model 1-3), OP1-4 (made by model 1-2) and OP1-5 (made by model 1-1), and thus the number of entries in meta-feature 1 in level-1 training set was equal to that of the initial training set. This procedure was repeated until all the level-0 algorithms were selected, and then there were n base models constructed, each of which consisted of five weak models and generated a meta-feature for the level-1 model (meta model). A meta model was trained on the out-of-fold predictions. With this meta model, the best combination of the base models according to the input data could be found. Test set was used to test the performance of the stacking meta model. In the test processes, base models were firstly used to predict the test set, and the outputs of each weak model in one base model would be combined by averaging (for regression model) or voting (for classification model) and used as the inputs for the meta model. The meta model then made predictions according to the outputs of the base models, and these predictions would be compared to the values in the initial test set to assess the performance of the stacking meta model.

Table S3. Optimized hyperparameters of the BG regression models, related to Star Methods

Models	Hyperparameters
SVR(rbf)	C=3, gamma=0.1
SVR(poly)	C=0.5, degree=4, gamma='auto'
SVR(linear)	C=1
LASSO	random_state=1, alpha=0.001
Ridge	random_state=1, alpha=1
KRR	alpha=0.01, kernel='rbf',gamma = 0.01
RF	n_estimators=100, max_depth=10, min_samples_split=3, min_samples_leaf=2
EXT	n_estimators=100, max_depth=10, min_samples_split=5, min_samples_leaf=3
GBR	n_estimators=100, max_depth=10, min_samples_split=10, min_samples_leaf=3
STR_{BG}	Meta_regressor**=** SVR(rbf), C=10, gamma=0.05

Table S4. Optimized hyperparameters of the H2 classification models, related to Star Methods

Models	MAE [eV]
RF	n_estimators=100, max_depth=20, min_samples_split=3, min_samples_leaf=2
EXT	n_estimators=100, max_depth=10, min_samples_leaf=2, min_samples_split=2
GBT	n_estimators=100, max_depth=10, min_samples_leaf=5, min_samples_split=2
Bagging(SVC-rbf)	C=5, gamma=0.01, n_estimators=100, max_samples=100
Bagging(SVC-poly)	C=10, degree=3, gamma='auto', n_estimators=100, max_samples=100
Bagging(SVC-linear)	C=0.1, n_estimators=100, max_samples=100
STC_{H2} **II**	Meta_classifier**=** EXT, n_estimators=100, max_depth=15, min_samples_leaf=5, min_samples_split=4

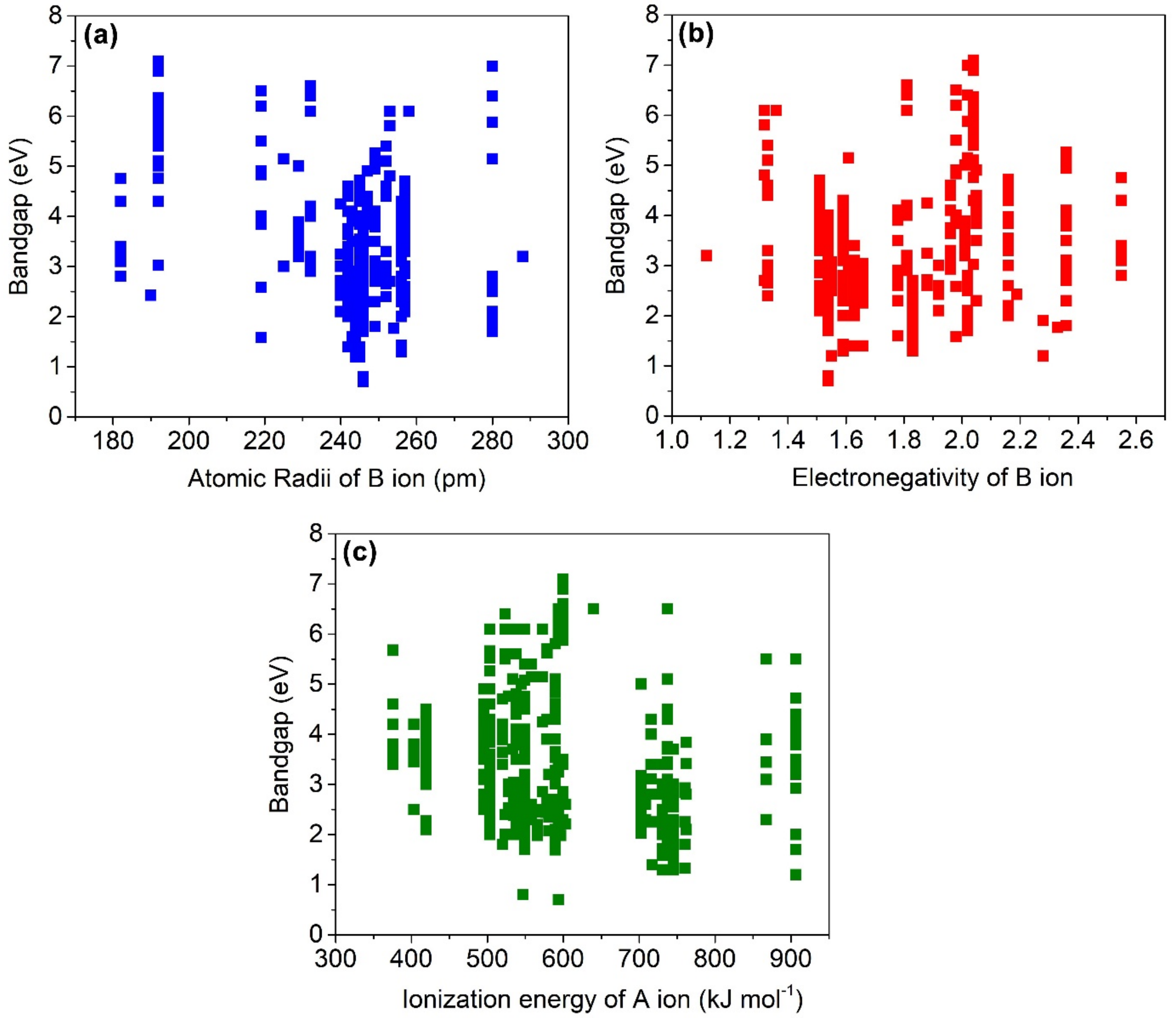

Figure S5. Three most important features: (a) R_B, (b) X_B and (c) E_A versus reported bandgap value of the photocatalysts in the bandgap dataset, related to Figure 3b.

Table S5. MAE and maximum error of the models in predicting the bandgap of the 10 samples in Table 2, related to Table 2.

Models	MAE [eV]	Maximum error [eV]
RF	0.73	-1.66
EXT	0.64	1.55
GBR	0.50	-1.57
KRR	0.54	-1.80
SVR(rbf)	0.50	-1.50
SVR(poly)	0.58	2.17
STR_{BG}	**0.17**	**-0.35**

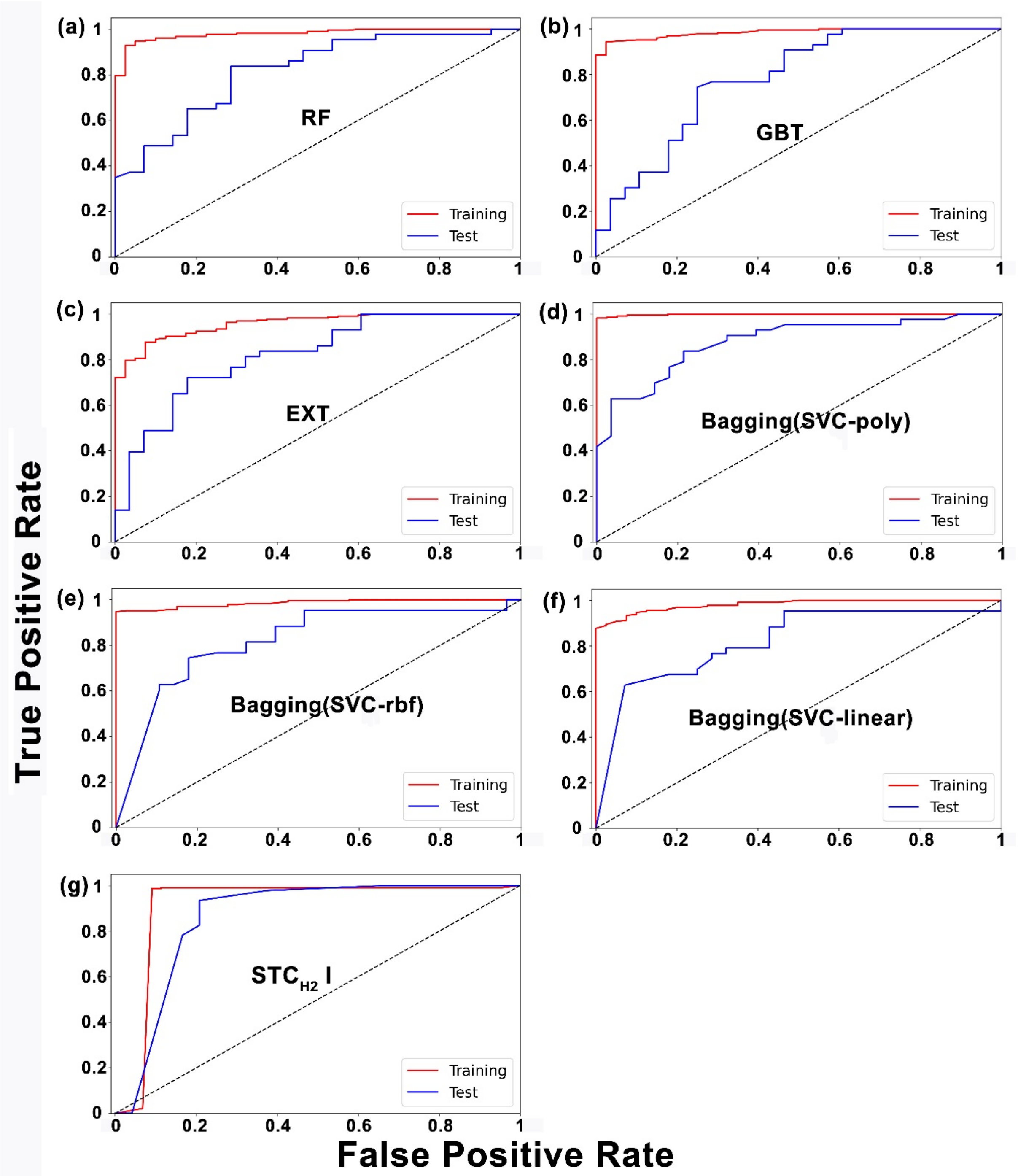

Figure S6. ROC (receiver operating characteristic) curves of the H_2 activity classification models without bandgap descriptor generated by (a) RF, (b) GBT, (c) EXT, (d) Bagging with 100 SVC(poly) weak classifiers (denoted as Bagging(SVC-poly)), (e) Bagging with 100 SVC(rbf) weak classifiers (denoted as Bagging(SVC-rbf)), (f) Bagging with 100 SVC(linear) weak classifiers (denoted as Bagging(SVC-linear)), and (g) Stacking algorithm (denoted as STC$_{H2}$ I), related to Table 3.

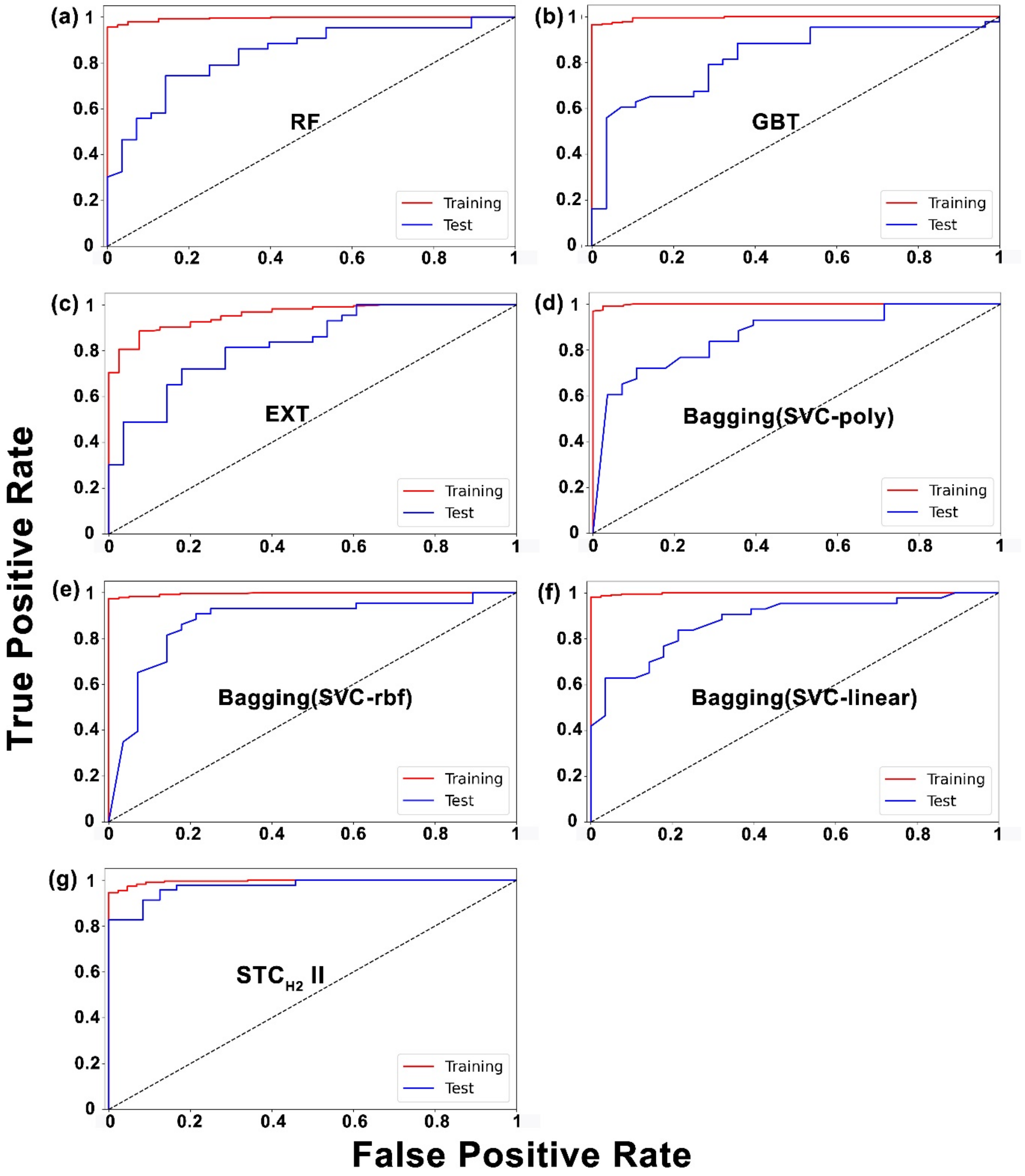

Figure S7. ROC curves of the H_2 activity classification models with bandgap descriptor generated by (a) RF, (b) GBT, (c) EXT, (d) Bagging with 100 SVC(poly) weak classifiers (denoted as Bagging(SVC-poly)), (e) Bagging with 100 SVC(rbf) weak classifiers (denoted as Bagging(SVC-rbf)), (f) Bagging with 100 SVC(linear) weak classifiers (denoted as Bagging(SVC-linear)), and (g) Stacking algorithm (denoted as STC_{H2} II), related to Table 4.

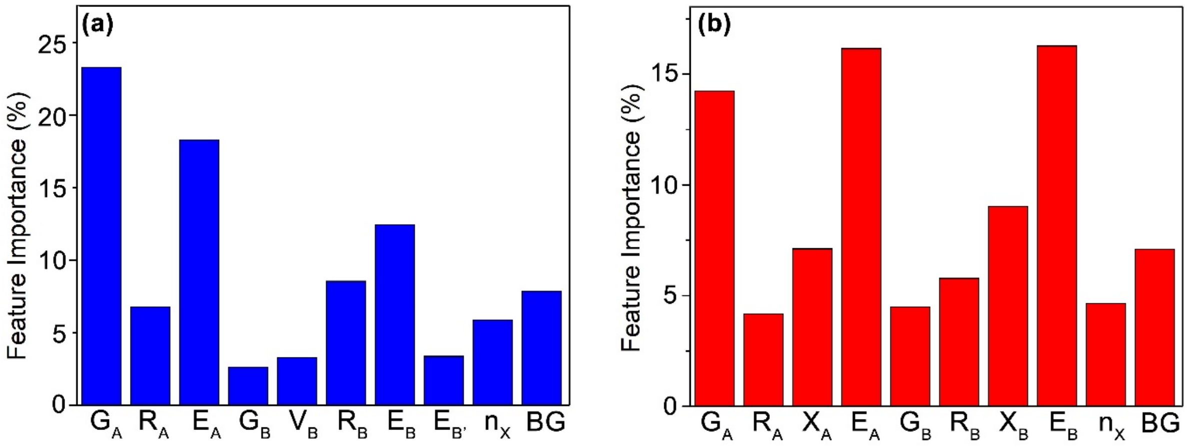

Figure S8. Relative importance of top 10 features evaluated by (a) GBT model and (b) RF model, related to Figure 4b. Among the three tree algorithms, RF exhibits the highest AUC, and GBT shows the highest F1 scores (Table 4). Therefore, we evaluate the relative importance of the features using the average of the importance calculated by RF (Figure S8a) and GBT (Figure S8b), shown in Figure 4b.

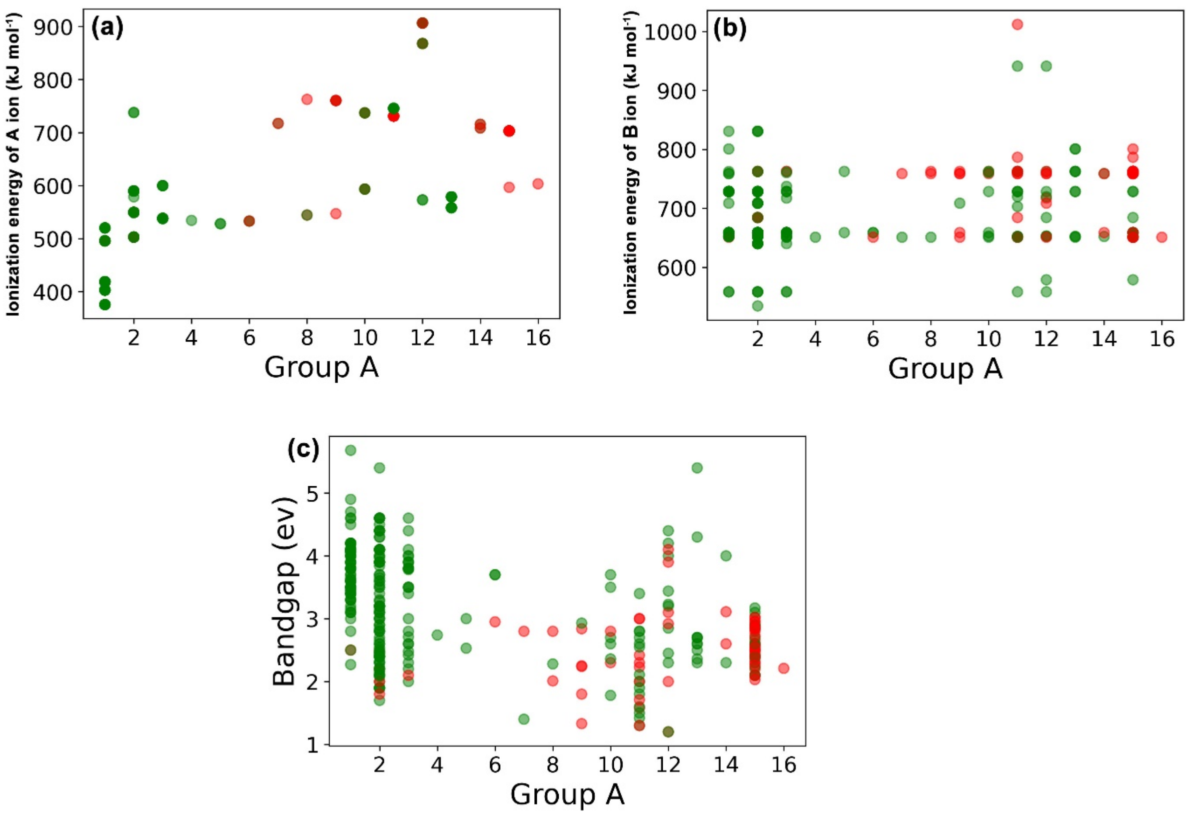

Figure S9. G_A versus (a) E_A, (b) E_B and (c) the reported bandgap value of the photocatalysts in H_2 dataset. The green dots denote H_2 active, and the red dots denote H_2 inactive, related to Figure 4b.

Table S6. 51 photocatalysts with predicted bandgap and H_2 evolution activity, related to Figure 5b.

Compounds	Predicted Bandgap [eV]	Predicted H_2 activity	Reported H_2 activity	Ref.
$NaVO_3$	3.25	0	0	(Konta *et al.*, 2003)
$GaTaO_3$	2.68	1	0	(Castelli *et al.*, 2013)
$Bi_{0.5}Y_{0.5}VO_4$	2.51	1	1	(Wang and Domen, 2020)
$SrGeO_3$	3.67	1	0	(Castelli *et al.*, 2013)
$Sr_{0.99}Cr_{0.01}Ti_{0.99}V_{0.01}O_3$	2.49	0	0	(Ishii *et al.*, 2004)
$KTa_{0.9}Ce_{0.1}O_{2.95}$	3.18	0	0	(Ishihara *et al.*, 1999)
$KTa_{0.9}Sb_{0.1}O_3$	2.91	0	0	(Ishihara *et al.*, 1999)
$K_2La_2Ti_{2.975}V_{0.025}O_{10.0125}$	3.26	0	0	(Yang *et al.*, 2009)
$Bi_{0.5}Eu_{0.5}VO_4$	2.55	1	1	(Wang and Domen, 2020)
$Bi_{0.5}Sm_{0.5}VO_4$	2.54	1	1	(Wang and Domen, 2020)
$Bi_{0.5}La_{0.5}VO_4$	2.59	1	1	(Wang and Domen, 2020)
$Bi_{0.5}Dy_{0.5}VO_4$	2.55	1	1	(Wang and Domen, 2020)
$Dy_2Ti_2O_7$	3.13	1	1	(Masanobu *et al.*, 2008)
$Ho_2Ti_2O_7$	3.01	1	1	(Masanobu *et al.*, 2008)
$Er_2Ti_2O_7$	3.03	1	1	(Masanobu *et al.*, 2008)
$Tm_2Ti_2O_7$	2.95	1	1	(Masanobu *et al.*, 2008)
$Eu_2Ti_2O_7$	3.14	1	1	(Masanobu *et al.*, 2008)
$Yb_2Ti_2O_7$	2.99	1	1	(Masanobu *et al.*, 2008)
$Lu_2Ti_2O_7$	3.05	1	1	(Masanobu *et al.*, 2008)
$Rb_2Ti_6O_{13}$	3.45	1	1	(Ogura *et al.*, 1997)
$SrNb_2O_6$	3.59	1	1	(Fujito *et al.*, 2016)
$LiVO_3$	3.34	0	0	(Castelli *et al.*, 2013)
$Sr_{0.99}Al_{0.01}TiO_{3.025}$	3.05	1	1	(Goto *et al.*, 2018)
$Sr_{0.99}Cr_{0.01}Ti_{0.99}Nb_{0.01}O_{3.01}$	2.55	1	1	(Ishii *et al.*, 2004)
$Sr_{0.99}Cr_{0.01}Ti_{0.99}Ta_{0.01}O_{3.01}$	2.54	1	1	(Ishii *et al.*, 2004)
$Sr_{0.98}Cr_{0.02}Ti_{0.99}Ta_{0.01}O_{3.015}$	2.52	1	1	(Ishii *et al.*, 2004)
$Sr_{0.9}Cr_{0.1}Ti_{0.9}Ta_{0.1}O_{3.1}$	2.51	1	1	(Ishii *et al.*, 2004)
$Ca_{0.96}Cu_{0.04}TiO_3$	2.43	1	1	(Zhang *et al.*, 2010)
$Ca_{0.92}(AgLa)_{0.04}TiO_3$	2.75	1	1	(Zhang *et al.*, 2012)
$BaTi_{0.99}Rh_{0.01}O_3$	2.19	1	1	(Maeda, 2014)
$BaTi_{0.95}Rh_{0.05}O_3$	2.26	1	1	(Maeda, 2014)
$Sr_{0.985}Li_{0.015}TiO_{2.9925}$	3.04	1	1	(Sakata et al., 2016)
$Sr_{0.985}K_{0.015}TiO_{2.9925}$	3.14	1	1	(Sakata *et al.*, 2016)
$Sr_{0.985}Rb_{0.015}TiO_{2.9925}$	3.15	1	1	(Sakata *et al.*, 2016)
$Sr_{0.985}Cs_{0.015}TiO_{2.9925}$	3.18	1	1	(Sakata *et al.*, 2016)
$Sr_{0.985}Mg_{0.015}TiO_3$	2.75	1	1	(Sakata *et al.*, 2016)
$Sr_{0.985}In_{0.015}TiO_{3.0075}$	2.66	1	1	(Sakata *et al.*, 2016)
$La_{1.9}Ba_{0.1}Ti_2O_{6.95}$	3.38	1	1	(Kim *et al.*, 2005)
$KTa_{0.9}Ga_{0.1}O_{2.9}$	2.96	1	1	(Ishihara *et al.*, 1999)
$KTa_{0.9}Hf_{0.1}O_{2.95}$	3.34	1	1	(Ishihara *et al.*, 1999)
$BaZr_{0.7}Sn_{0.3}O_3$	3.35	1	1	(Yuan *et al.*, 2010)
$Bi_2Ga_{3.6}Fe_{0.4}O_9$	2.86	1	1	(Yang *et al.*, 2017)
$Bi_{0.5}Nd_{0.5}VO_4$	2.56	1	1	(Wang and Domen, 2020)
$Bi_{0.5}Gd_{0.5}VO_4$	2.54	1	1	(Wang and Domen, 2020)
$SrTi_{0.985}Rh_{0.005}Sb_{0.01}O_{3.005}$	2.65	1	1	(Asai *et al.*, 2014)
$Sr_{0.93}Ba_{0.07}In_2O_4$	2.86	1	1	(Sato *et al.*, 2003)
$K_2La_2Ti_{2.985}V_{0.015}O_{10.0075}$	3.25	0	1	(Yang *et al.*, 2009)
$La_{1.9}Ca_{0.1}Ti_2O_{6.95}$	3.13	1	1	(Kim *et al.*, 2005)
$La_{1.9}Sr_{0.1}Ti_2O_{6.95}$	3.35	1	1	(Kim *et al.*, 2005)
$InNbO_4$[a)]	2.68	1	1	(Zhou *et al.*, 2017)
$Li_{0.93}Cu_{0.07}Nb_3O_8$[b)]	1.99	1	1	(Sahoo and Maggard, 2013)

a) Samples synthesized by different methods show different bandgap

b) Two bandgap values are found in terms of the UV-vis absorbance spectrum

Table S7. 20 candidates with predicted bandgap and H_2 evolution activity discovered by the multi-step target-driven approach, related to Figure 6.

Compound	Predicted Bandgap [eV]	Predicted H_2 activity
$Ag_2Ti_6O_{13}$	2.76	1
Ba_3CrFeO_8	2.64	1
$Ba_{1.8}La_{0.2}Ti_6O_{14.1}$	2.72	1
$BiLaTi_4O_{11}$	2.76	1
$Bi_5YTi_3Fe_2O_{18}$	2.41	1
$Bi_9Ti_6FeO_{27}$	2.73	1
$Ca_2Fe_2O_5$	2.24	1
$Ca_2Mn_3O_8$	2.70	1
Co_2TiO_4	1.96	1
$Co_2SrV_2O_8$	2.18	1
$Cr_2V_4O_{13}$	2.54	1
$Cu_2Ti_6O_{13}$	2.80	1
$Cu_{0.5}Co_{0.5}Fe_2O_4$	2.04	1
$CuTi_2O_5$	2.30	1
$Cu_{0.5}Ca_{0.5}Fe_2O_4$	1.95	1
$Fe_{0.5}In_{0.5}VO_4$	2.46	1
$KBiFe_2O_5$	1.88	1
$Ni_2V_2O_7$	2.45	1
$Sr_2BiV_3O_{11}$	2.66	1
$SrCrFeO_4$	2.55	1

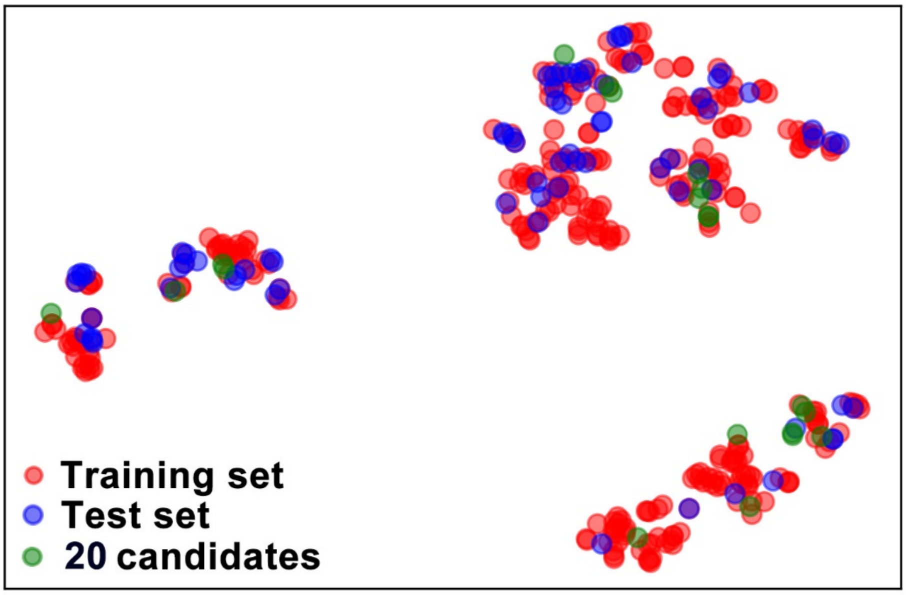

Figure S10. Reduced two-dimensional feature space of the training set (red), test set (blue) and the 20 candidates selected by ML models (green) obtained by the t-SNE algorithm, related to Figure 6.

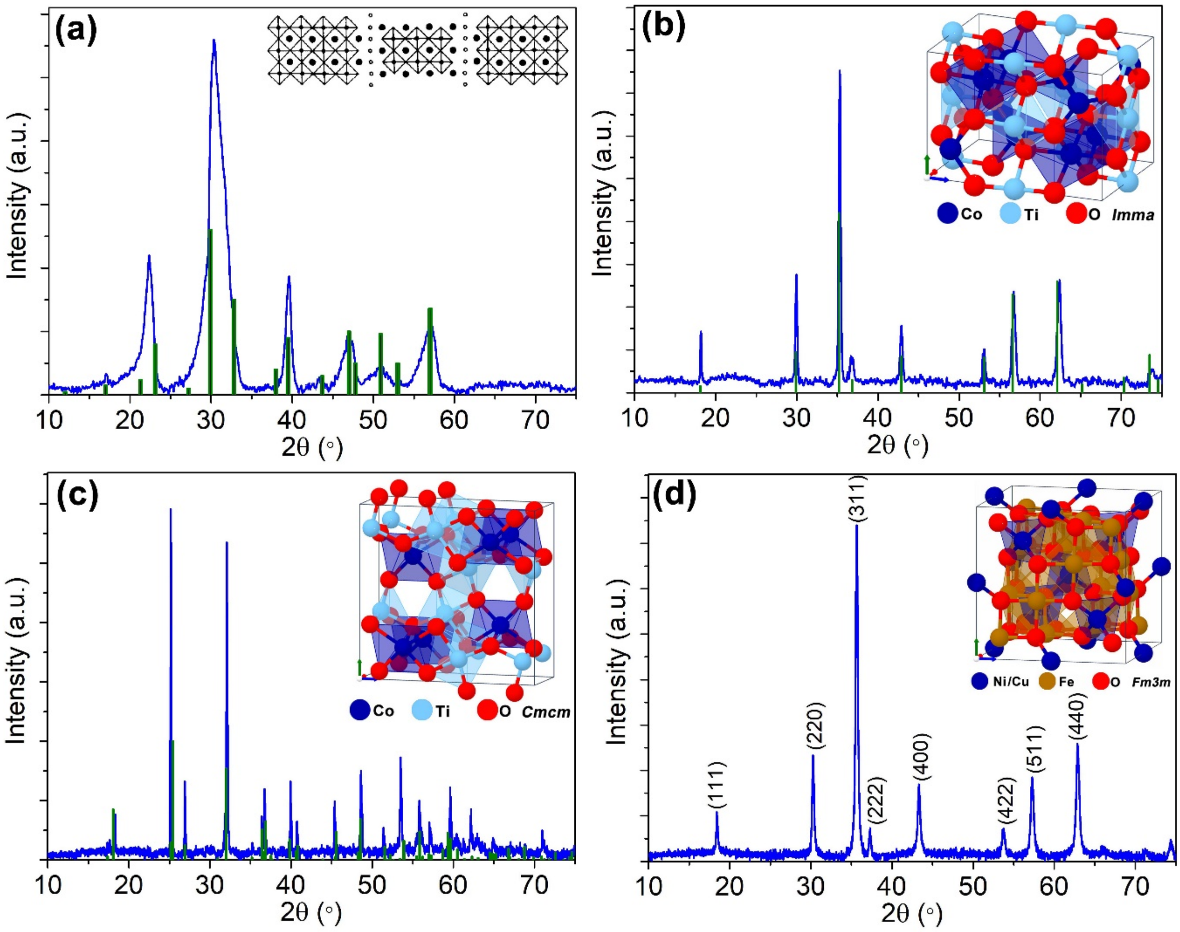

Figure S11. XRD patterns of (a) $Bi_9Ti_6FeO_{27}$ (green lines referred to JCPDS #42-0052, layered perovskite (Subbanna *et al.*, 1990)), (b) Co_2TiO_4 (green lines referred to JCPDS #39-1410, inverse spinel (Khanahmadzadeh *et al.*, 2015)), (c) $CoTi_2O_5$ (green lines referred to The Materials Project #mp-753066, pseudobrookite (Anderson *et al.*, 2019)), and (d) $Cu_{0.5}Ni_{0.5}Fe_2O_4$ (the indexation is according to JCPDS #54-0964 and JCPDS #01-077-0010, spinel (Velinov *et al.*, 2013)). Insets are the corresponding crystal structures, related to Figure 7.

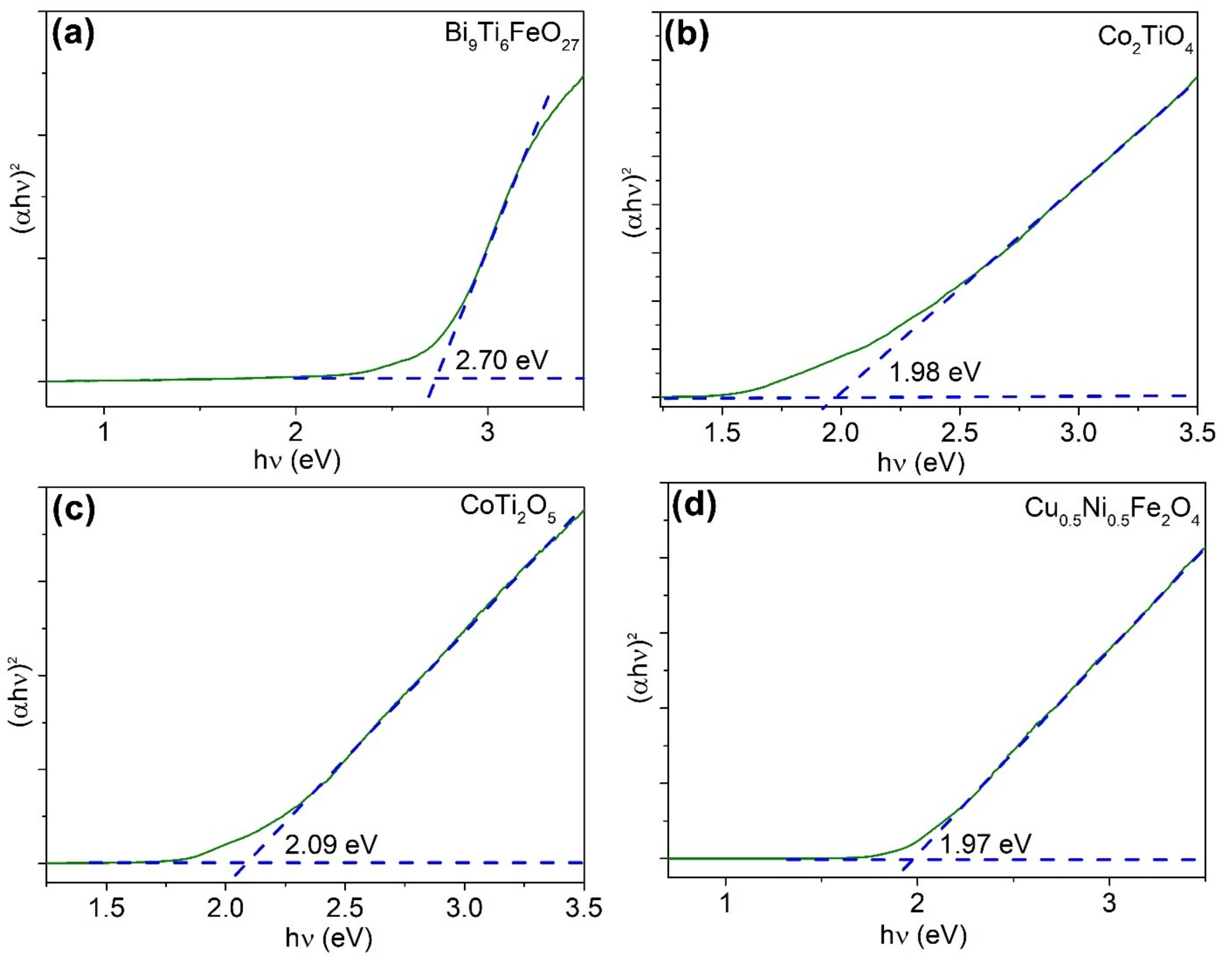

Figure S12. Bandgap of (a) $Bi_9Ti_6FeO_{27}$, (b) Co_2TiO_4, (c) $CoTi_2O_5$, and (d) $Cu_{0.5}Ni_{0.5}Fe_2O_4$, obtained from the intersection of the tangents of the curve and the baseline of the Tauc plots shown as blue dashed lines, related to Figure 7a.

Table S8. Predictions of the bandgap of the 4 compounds selected by the ML models, related to Figure 7a.

Compound	Predicted Bandgap [eV]	Experimental Bandgap [eV]	Error [eV]
$Bi_9Ti_6FeO_{27}$	2.73	2.70	+0.03
Co_2TiO_4	1.96	1.98	-0.02
$CoTi_2O_5$	2.03	2.09	-0.06
$Cu_{0.5}Ni_{0.5}Fe_2O_4$	2.00	1.97	+0.03

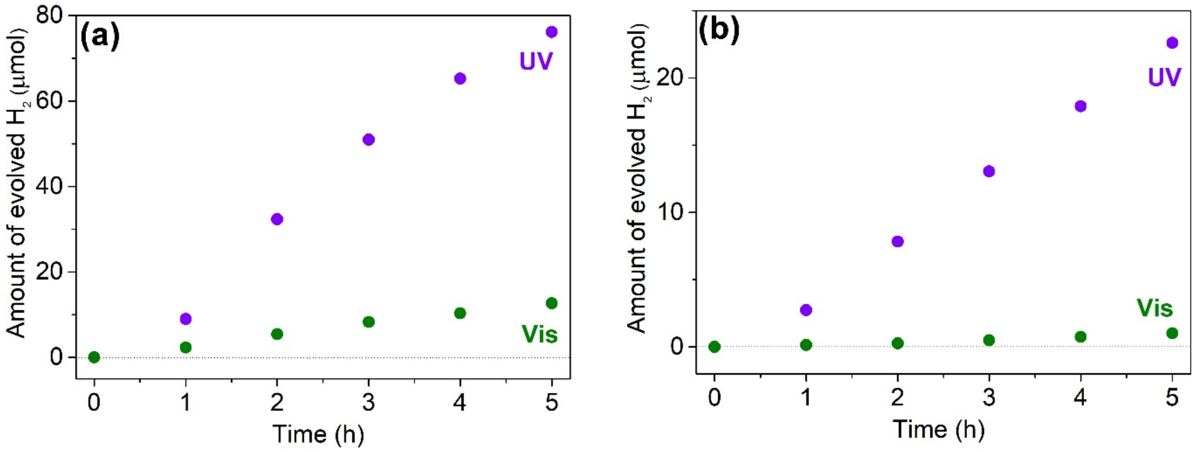

Figure S13. Time course of water splitting on (a) $Bi_9Ti_6FeO_{27}$ and (b) Co_2TiO_4 in aqueous methanol solution (10 vol%) under UV (purple dots) and visible (green dots) illumination from a xenon lamp (UV, $\lambda > 300$ nm; visible, 400 nm $< \lambda <$ 800 nm), related to Figure 7b.
